# RGS20 Promotes Tumor Progression through Modulating PI3K/AKT Signaling Activation in Penile Cancer

**DOI:** 10.1155/2022/1293622

**Published:** 2022-04-19

**Authors:** Dazun Shi, Shiyu Tong, Hui Han, Xiheng Hu

**Affiliations:** ^1^Department of Gynecology, Xiangya Hospital, Central South University, Changsha, China; ^2^Department of Urology, Xiangya Hospital, Central South University, Changsha, China; ^3^Department of Urology, Sun Yat-sen University Cancer Center, Guangzhou, China; ^4^Department of Dermatology, The Hunan Engineering Research Center of Skin Heath and Disease, Xiangya Hospital, Central South University, China

## Abstract

Regulator of G protein signaling 20 (RGS20) plays an important role in regulating neuronal G protein-coupled receptor signaling; however, its expression and oncogenic function in penile cancer (PC) remains unclear. Here, we observed high RGS20 expression in PC tissues compared to normal/adjacent penile tissues, which was closely associated with tumor stage, nodal status, and pelvic metastasis in our PC cohort. The cellular functional analysis of RGS20 revealed that manipulation of the RGS20 expression markedly affected cell viability, BrdU incorporation, soft agar clonogenesis, caspase-3 activity, and cell migration/invasion in PC cell models. Moreover, RGS20 could interact with PI3K p85*α* subunit and regulate PI3K/AKT signaling activation in PC cell lines. Knockdown of the PI3K p85*α* or p110*α* subunit attenuated cell viability, BrdU incorporation, soft agar clonogenesis, and cell migration/invasion in PC cell lines. In contrast, the overexpression of constitutively activated PI3K p110*α* mutant restored cell proliferation and cell migration/invasion caused by RGS20 depletion in PC cells. Consistent with the in vitro findings, RGS20 depletion attenuated PI3K/AKT signaling activation and suppressed tumor growth in a murine xenograft model. Importantly, the high RGS20 expression was associated with PI3K/AKT signaling activation and unfavorable progression-free/overall survival, highlighting the clinical relevance of RGS20/PI3K/AKT signaling in PC. In conclusion, the aberrant RGS20 expression may serve as a diagnostic and prognostic marker for PC. RGS20 may promote PC progression through modulating PI3K/AKT signaling activation, which may assist with the development of RGS20-targeting therapeutics in the future.

## 1. Introduction

Although penile cancer (PC) is rare in developed countries (0.5–1.6 per 100,000 men), its incidence is thought to be much higher in developing countries of South America, Africa, and Asia [1]. Currently, surgery is the main treatment for PC; although, chemotherapy, targeted therapy, and brachytherapy are also applied [2, 3]. Clinical parameters, including human papillomavirus (HPV) status, histological subtype, pathological grade, and clinical stage, have been demonstrated to be closely related to the clinical outcome of PC [4]. Despite considerable progress in clinical treatment, the overall clinical outcome of PC has not been improved during the last 20 years [5]. A challenge in developing novel strategies for PC is the limited understanding of the molecular mechanisms driving PC carcinogenesis and tumor progression. Recently, many genes and signaling pathways, including HPV [6], *β*-Catenin [7], Id1 [8], tumor suppressors p53 and p16 [9], and PI3K/AKT/mTOR [10], have been shown to exert important oncogenic/tumor suppressor function in PC. Further understanding of the molecular mechanisms of PC carcinogenesis may assist with the development of novel therapeutic strategies for PC.

Regulators of G protein signaling genes (RGSs) are key regulators of G protein-coupled receptor (GPCR) signaling in a diverse range of organisms [11]. RGS proteins act as negative regulators of G-protein signaling by binding to and enhancing GTP hydrolysis by G-protein *α* subunits. Until now, at least 20 RGS genes have been identified, with distinct functions in modulating neuronal GPCR signaling, including dopamine and opioid receptor signaling. Recently, increasing evidence suggests that RGSs may also function as tumor suppressors or oncogenes in many cancers. RGS2 and RGS4 are proposed to be tumor suppressors and may suppress breast cancer cell growth [12, 13]. Moreover, RGS5 deficiency has been reported to be associated with tumor progression in lung cancer [14], while RGS6 exerts its tumor suppressor function via G protein-independent signaling mechanisms in breast cancer [15]. Furthermore, RGS17 is upregulated and promotes tumor growth and migration in lung and prostate cancer [16]. However, the expression and clinical significance of RGSs in PC still remain unknown.

The RGS20 gene is located at human chromosome 8q11.23, which encodes a member of the RGS protein family. As a RGS RZ family member, RGS20 shares about 62% similarity with RGS17, and they have the same cysteine-rich motif in the N-terminal domain [17]. RGS20 regulates neuronal G protein-coupled G*α* (i) receptor signaling in the brain [18]. Recently, the dysregulated RGS20 expression has been documented in breast, bladder, and renal cancer [19–21]. However, the expression and function of RGS20 in PC remain unclear. Our preliminary analysis on GSE57955 dataset showed that RGS20 was the top RGSs highly expressed in PC. Therefore, we next explored the oncogenic function of RGS20 in PC cell models in vitro and in vivo. Our findings suggested that RGS20 serves as an oncogene through modulating PI3K/AKT signaling activation in PC, which may assist with the development of RGS20-targeting therapeutics in the future.

## 2. Materials and Methods

### 2.1. Patient and Tumor Characteristics

Patients with PC who had undergone surgery and were diagnosed with PC between 2013 and 2017 were recruited. These patients had no prior history of clinical treatment before surgery. The TNM stage of the patients was assigned according to the updated 8th edition AJCC TNM staging system [22]. The study protocols were approved by the institutional research ethics committee (Rev No. 201805847). Clinical follow-up was conducted to monitor cancer/vital status at our institution.

### 2.2. Reagents and Cell Lines

Primary antibodies were purchased from the following sources: RGS20 antibody (Invitrogen); AKT, p-AKT (T308), p-AKT (S473), PI3K (p85*α*), PI3K (p110*α*), and *β*-actin antibodies (Cell Signaling Technology). The human PC cell lines Penl1, 149RM, 149RCa, and LM156 were established and routinely cultured as described previously [23]. Lentiviruses encoding shRNAs targeting RGS20 and PI3K p85*α*/p110*α* were obtained from Genecopoeia, as were LV105 lentiviruses encoding empty vector (EV) or RGS20. HA-tagged pCMV3 plasmid encoding empty vector (EV) or PI3K p110*α* was purchased from Sinobiological (Beijing, China). The PIK3CA/p110*α* dead mutant (D933A) and constitutively active mutant (myristoylated form, Myr) were constructed using QuikChange Lightning Site-Directed Mutagenesis Kit (Agilent, Santa Clara, CA).

### 2.3. Cell Viability Analysis

Cell viability was evaluated using a CCK-8 assay kit according to the user's manual. Briefly, PC cells were plated in 96-well culture plates in triplicate (2 × 10^3^ cells/well). Cell viability was measured 96 h after cell plating. The CCK-8 absorbance (OD_450_) was measured by a Multiskan MK3 microplate reader.

### 2.4. Soft Agar Assay

The in vitro clonogenic potential of PC cells was evaluated using a soft agar assay as we described previously [24]. Briefly, a 1.5 mL culture medium with 0.5% agar was plated into each well of a 6-well culture plate. After the agar had solidified, each well received another 1.5 mL of 0.35% agar in a culture medium containing 5 × 10^3^ PC cells. After 10-12 days, the colonies in each group were counted.

### 2.5. PI3K Activity Assay

The PI3K activity was determined by measuring the level of PI (3, 4, 5) and P_3_ (PIP3) converted from PI3K substrate PI (4, 5) and P_2_ (PIP2). A PI3K activity ELISA kit (Echelon Biosciences, Salt Lake City, UT) was used according to the user's instructions.

### 2.6. Caspase-3 Activity Assay

A caspase-3 colorimetric assay kit (Abcam, Waltham, MA) was used to quantify the caspase-3 activity. Briefly, PC cells (1 × 10^6^ cells) were harvested with lysis buffer. Protein lysate was mixed with assay buffer containing caspase-3 substrate Ac-DEVD-pNA and was further incubated for 1 h at 37°C. The absorbance (OD_405_) of the resulting product pNA was measured using a Thermo MK3 microplate reader.

### 2.7. Subcellular Fractionation Analysis

A NE-PER nuclear and cytoplasmic extraction kit (Thermo Fisher Scientific) was used to fractionate proteins into nuclear and cytoplasmic fractions according to the manufacturer's protocol. Protein concentration was estimated, and the samples were boiled with 2× SDS sample loading buffer.

### 2.8. Coimmunoprecipitation

Coimmunoprecipitation of RGS20 was conducted as we described previously [25]. RGS20 was immunoprecipitated using a rabbit anti-RGS20 antibody and captured by Protein G Dynabeads. The resulting immunoprecipitates were analyzed by western blotting.

### 2.9. Western Blotting

Total cellular proteins were extracted by RIPA (radio immunoprecipitation assay) lysis buffer. Protein lysate (20 *μ*g protein) was separated by sodium dodecyl sulfate polyacrylamide gel electrophoresis (SDS-PAGE) and transferred to Polyvinylidene fluoride (PVDF) membranes. Protein blots were detected by antigen-antibody reaction and were visualized using a Millipore ECL substrate kit.

### 2.10. Immunohistochemistry (IHC)

Archival paraffin-embedded PC tissues were collected for immunohistochemistry. The tissue sections were dewaxed, rehydrated, and subjected to heat-induced antigen retrieval. Antigen-antibody reactions (Antibody dilution for RGS20: 1 : 100; dilution for p-AKT: 1 : 200) were visualized by exposure to the chromogen substrate. The scoring criteria for the IHC results described by Tan et al. [26] were employed. An IHC score ≥ 4 was considered to indicate high expression.

### 2.11. RNA Sequencing

The mRNA was extracted from Penl1 cells with or without RGS20 knockdown. The mRNA libraries were constructed, and RNA sequencing was performed on an Illumina NovaSeq6000 platform (HaploX, Shenzhen, China). The expression level of protein-coding genes was calculated as fragments per kilobase of the exon model per million mapped fragments (FPKM) value.

### 2.12. Pathway and Process Enrichment Analysis

Pathway and process enrichment analysis was conducted in *Metascape* (http://metascape.org/gp/index.html), with module sources including GO process, Reactome, and KEGG pathway [27]. *P* values were calculated based on the accumulative hypergeometric distribution.

### 2.13. Gene Expression Omnibus (GEO) Dataset

The GEO dataset GSE57955 can be downloaded from the NCBI GEO website (https://www.ncbi.nlm.nih.gov/geo/query/acc.cgi?acc=GSE57955). Gene expression data were analyzed as described previously [28]. Genes with a mean log_2_ signal ratio (PC/normal penile tissue pool) of ≥1.0 and ≤ −1.0 were considered differentially expressed.

### 2.14. Gene Set Enrichment Analysis

The GSE57955 dataset was used for gene set enrichment analysis (GSEA). Gene expression profiles were compared between RGS20-high (*n* = 20) and RGS20-low PC (*n* = 19) based on the enrichment of KEGG pathway signatures [29]. The nominal *P* value (NOM *P* val) and normalized enrichment score (NES) were calculated. A nominal *P* < 0.05 was considered significant in this analysis.

### 2.15. Animal Studies

For the in vivo studies, Penl1 cells with or without RGS20 knockdown were inoculated subcutaneously into the right flank (1 × 10^6^ cells/mouse, *n* = 6). The greatest longitudinal diameter (a) and the greatest transverse diameter (b) of the subcutaneous tumor were recorded. Tumor volume was calculated using the modified ellipsoidal formula: tumor volume (mm^3^) = *a* × *b*^2^/2. Mice were sacrificed 26 d after PC cell inoculation, and the subcutaneous xenografts were removed and weighed. The expression of RGS20, Ki-67, p-AKT (T308/S473), and cleaved caspase-3 in xenograft tissues was evaluated by IHC and western blotting.

### 2.16. Statistical Analysis

SPSS software (version 16) was used for statistical analysis. The differences in RGS20 expression in paired and PC specimens were examined by the Wilcoxon rank sum test. The relationship between the RGS20 expression and clinicopathological parameters was analyzed by the chi-square test. The means of the two groups were compared by two-tailed unpaired Student's *t*-test with Welch's corrections one-way ANOVA followed by Dunnett's multiple comparisons test was used to compare the means of three or more groups. Progression-free survival and overall survival in RGS20-high and RGS20-low PC groups were compared by the log-rank test. For statistical analysis, *P* < 0.05 (two-tailed) was considered significant.

### 2.17. Supplementary Methods

Experimental procedures regarding lentiviral particle packaging, BrdU incorporation assay, wound-healing assay, and transwell invasion assay can be found in the supplementary files (available here).

## 3. Results

### 3.1. The aberrant RGS20 Expression Is Correlated with Tumor Progression and Unfavorable Clinical Outcome

**Figure 1:**
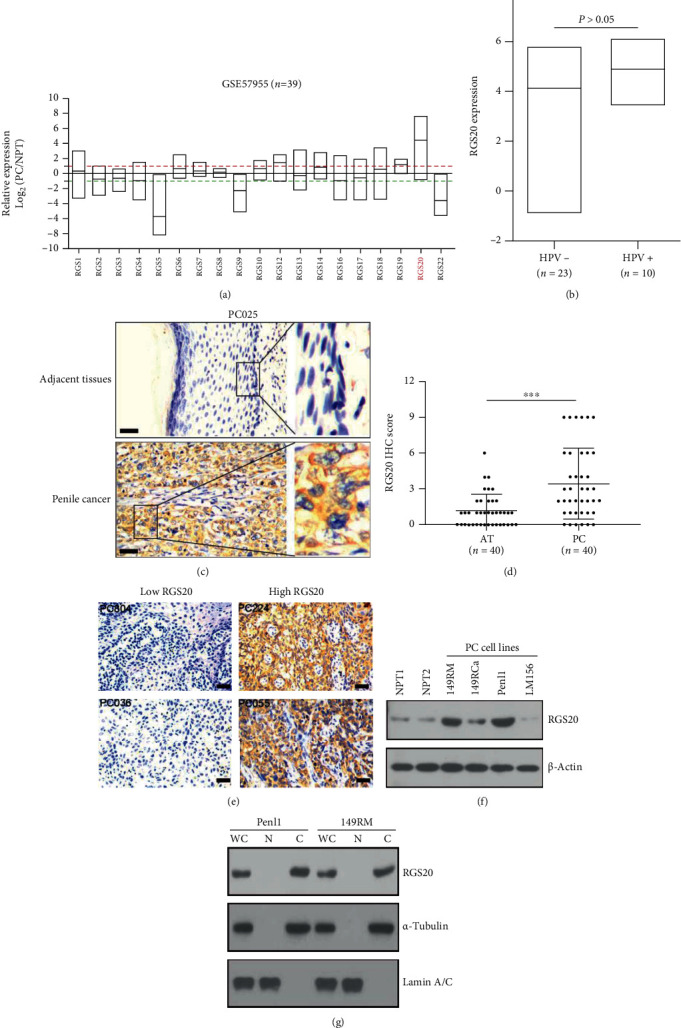
RGS20 is highly expressed in PC and associated with unfavorable progression-free survival. (a) The relative expression of RGSs in GSE57955 dataset (*n* = 39). The relative RGS expression was calculated with reference to normal penile tissue pool. (b) The RGS20 expression was not associated with HPV infection in GSE57955 dataset. HPV^+^ cases vs. HPV^−^ cases, *P* > 0.05. (c) Immunohistochemical staining showed the RGS20 expression in paired adjacent penile tissues or PC tissues, respectively. Bars: 50 *μ*m. (d) Immunohistochemical staining showed that RGS20 was highly expressed in PC tissues compared to paired adjacent penile tissues (*n* = 40). (e) Immunohistochemical staining showed high or low RGS20 expression in PC tissues, bars: 50 *μ*m. (f). Western blotting analysis on the expression of RGS20 in normal penile tissues (NPT1, NPT2) and a panel of PC cell lines (149RM, 149RCa, Penl1, and LM156). *β*-Actin was used as loading control. (g) Subcellular fractionation analysis showed that RGS20 was mostly present in the cytoplasmic fraction of PC cells. N: nuclear fraction; C: cytoplasmic fraction; WCL: whole cell lysate.

The expression of RGSs in the GSE57955 dataset was extracted and analyzed with reference to normal penile tissues. RGSs exhibited different expression patterns in PC and normal penile tissues, with RGS20 exhibiting the highest expression among RGSs in PC (mean log_2_ (PC/NPT) = 4.495) (Figure 1(a)). In contrast, the expression of RGS5, RGS9, and RGS22 was relatively low in PC (Figure 1(a)). Although the RGS20 expression in HPV+ cases seemed to be higher than that in HPV– cases, the difference was not statistically significant (Figure 1(b)). We next examined the RGS20 expression in forty paired adjacent tissues (AT) and PC using IHC. The RGS20 expression in AT was markedly lower than that in paired PC tissues (Figures 1(c) and 1(d)). We also evaluated the expression of RGS20 in our PC cohort, with 40.4% (38/94) of PC cases exhibiting the high RGS20 expression (IHC score ≥ 4) (Figure 1(e), Table 1). The high RGS20 expression was significantly related to tumor (*T*) stage (*P* = 0.028), nodal status (*P* = 0.002), and pelvic metastasis (*P* = 0.019), but not to phimosis (*P* = 0.106), body mass index (*P* = 0.347), age (*P* = 0.502), histological subtype (*P* = 0.101), or pathological grades (*P* = 0.379) (Table 1). Western blotting analysis showed high RGS20 expression in Penl1 and 149RM compared to 149RCa, LM156, and normal penile tissues (NPT1, NPT2) (Figure 1(f)). Subcellular fractionation analysis showed that the RGS20 protein is mostly present in the cytoplasm (Figure 1(g)).

### 3.2. Knockdown of the RGS20 Expression Suppresses Malignant Phenotypes in RGS20-High PC Cell Lines

**Figure 2:**
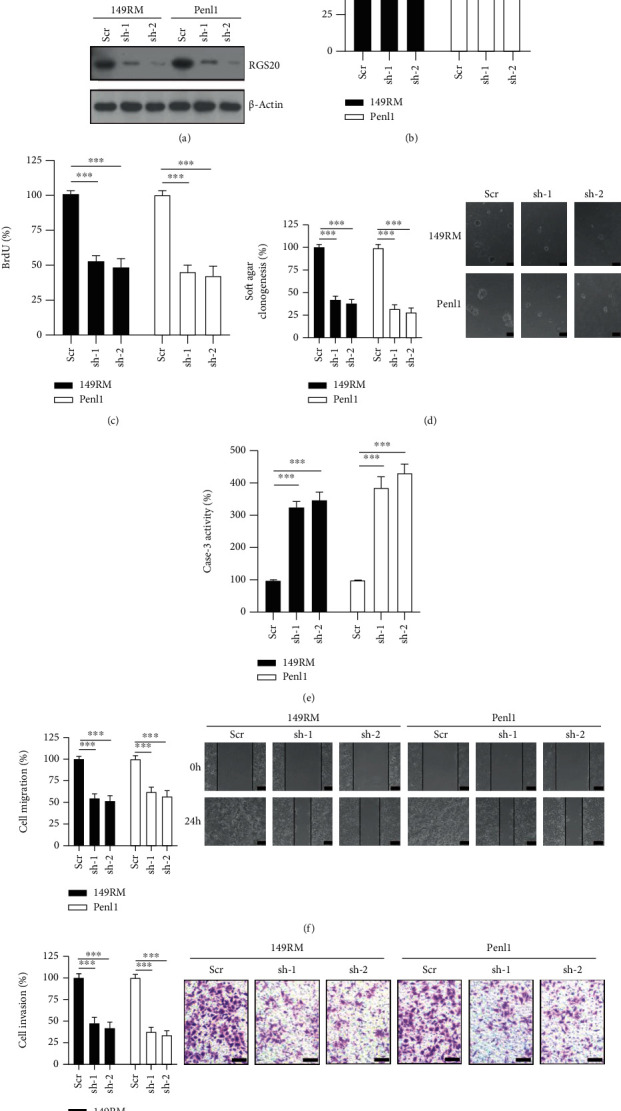
Knockdown of RGS20 suppresses cell proliferation, soft agar clonogenesis, and migration/invasion in PC cell lines. (a) Western blotting on RGS20 expression in 149RM and Penl1 cells transfected with scramble (Scr) control or RGS20 knockdown lentiviruses. *β*-Actin was used as loading control. (b) CCK-8 analysis on cell viability following RGS20 knockdown in 149RM and Penl1 cells. The cell viability in Scr control was regards as 100%. *n* = 4, ^∗∗∗^*P* < 0.001. (c) BrdU incorporation analysis on cell proliferation following RGS20 knockdown in 149RM and Penl1 cells. The BrdU incorporation in Scr control was regards as 100%. *n* = 4, ^∗∗∗^*P* < 0.001. (d) Soft agar clonogenesis of PC cells following RGS20 knockdown. The soft agar clonogenesis in Scr control was regards as 100%. *n* = 4, ^∗∗∗^*P* < 0.001. Bars: 100 *μ*m. (e) Caspase-3 activity of PC cells following RGS20 knockdown. The caspase-3 activity in Scr control was regards as 100%. *n* = 4, ^∗∗∗^*P* < 0.001. (f) Wound healing assay on PC cells following RGS20 knockdown. Bars: 100 *μ*m. The cell migration in Scr control was regards as 100%. *n* = 4, ^∗∗∗^*P* < 0.001. (g) Transwell invasion assay on PC cells following RGS20 knockdown. Bars: 100 *μ*m. The cell invasion in Scr control was regards as 100%. *n* = 4, ^∗∗∗^*P* < 0.001.

We generated PC cell lines with RGS20 knockdown to study the cellular function of RGS20 in PC. The addition of RGS20-specific shRNA lentivirus greatly reduced the RGS20 expression in 149RM and Penl1 cells (Figure 2(a)). RGS20 knockdown reduced cell viability and BrdU incorporation compared to the scramble (Scr) control in 149RM and Penl1 (Figures 2(b) and 2(c)). RGS20 knockdown also greatly increased caspase-3 activity and impaired soft agar clonogenesis compared to Scr in 149RM and Penl1 (Figures 2(d) and 2(e)). Furthermore, cell migration and invasion were significantly attenuated in the shRGS20 group compared to Scr in 149RM and Penl1 (Figures 2(f), 2(g)). As both RGS20 shRNAs exhibited a similar inhibitory effect on PC cell models, RGS20 sh-2 shRNA was selected for further experiments.

### 3.3. The Overexpression of RGS20 Promotes Malignant Phenotypes in RGS20-Low PC Cell Lines

**Figure 3:**
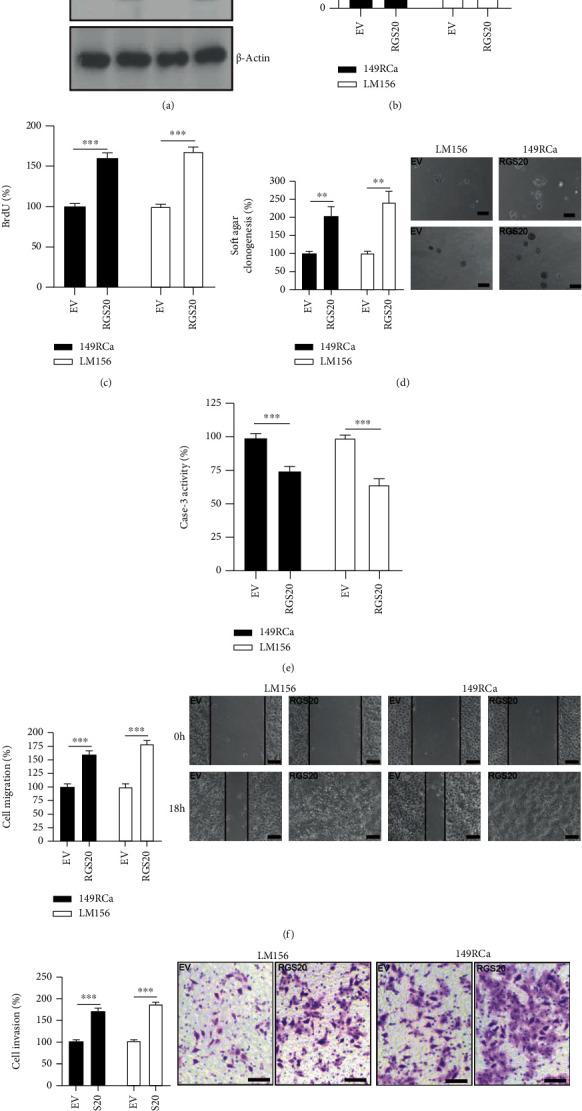
The overexpression of RGS20 enhances cell proliferation, soft agar clonogenesis, and migration/invasion in RGS20-low PC cell lines. (a) Western blotting on the RGS20 expression in 149RCa and LM156 cells transfected with empty vector (EV) or RGS20 lentiviruses. *β*-Actin was used as loading control. (b) CCK-8 analysis on cell viability following the RGS20 overexpression in PC cells. The cell viability in EV control was regards as 100%. *n* = 4, ^∗∗∗^*P* < 0.001. (c) BrdU incorporation analysis on cell proliferation following the RGS20 overexpression in PC cells. The BrdU incorporation in EV control was regards as 100%. *n* = 4, ^∗∗∗^*P* < 0.001. (d) Soft agar clonogenesis of PC cells following the RGS20 overexpression in PC cells. The soft agar clonogenesis in EV control was regards as 100%. *n* = 4, ^∗∗^*P* < 0.01. Bars: 100 *μ*m. (e) Caspase-3 activity of PC cells following the RGS20 overexpression. The caspase-3 activity in EV control was regards as 100%. *n* = 4, ^∗∗∗^*P* < 0.001. (f) Wound healing assay on PC cells following the RGS20 overexpression. Bars: 100 *μ*m. The cell migration in EV control was regards as 100%. *n* = 4, ^∗∗∗^*P* < 0.001. (g) Transwell invasion assay on PC cells following the RGS20 overexpression. Bars: 50 *μ*m. The cell invasion in EV control was regards as 100%. *n* = 4, ^∗∗∗^*P* < 0.001.

The function of RGS20 was also explored in the RGS20-low PC cell lines, LM156, and 149RCa. Transfection with RGS20 lentiviruses greatly increased RGS20 expression compared to empty vector (EV) control in LM156 and 149Rca cells (Figure 3(a)). RGS20-expressing PC cells grew faster than the EV control, which was accompanied by increased BrdU incorporation in LM156 and 149RCa cells (Figures 3(b) and 3(c)). Soft agar clonogenesis of PC cells in the RGS20 group was increased greatly compared to that in the EV control (Figure 3(d)). Additionally, the activity of caspase-3 was decreased in the RGS20 group compared to the EV group (Figure 3(e)). Furthermore, the overexpression of RGS20 markedly accelerated cell migration and invasion, as compared to that observed in the EV control in LM156 and 149RCa cells (Figures 3(f) and 3(g)).

### 3.4. RGS20 Regulates PI3K/AKT Signaling Activation in PC Cells

**Figure 4:**
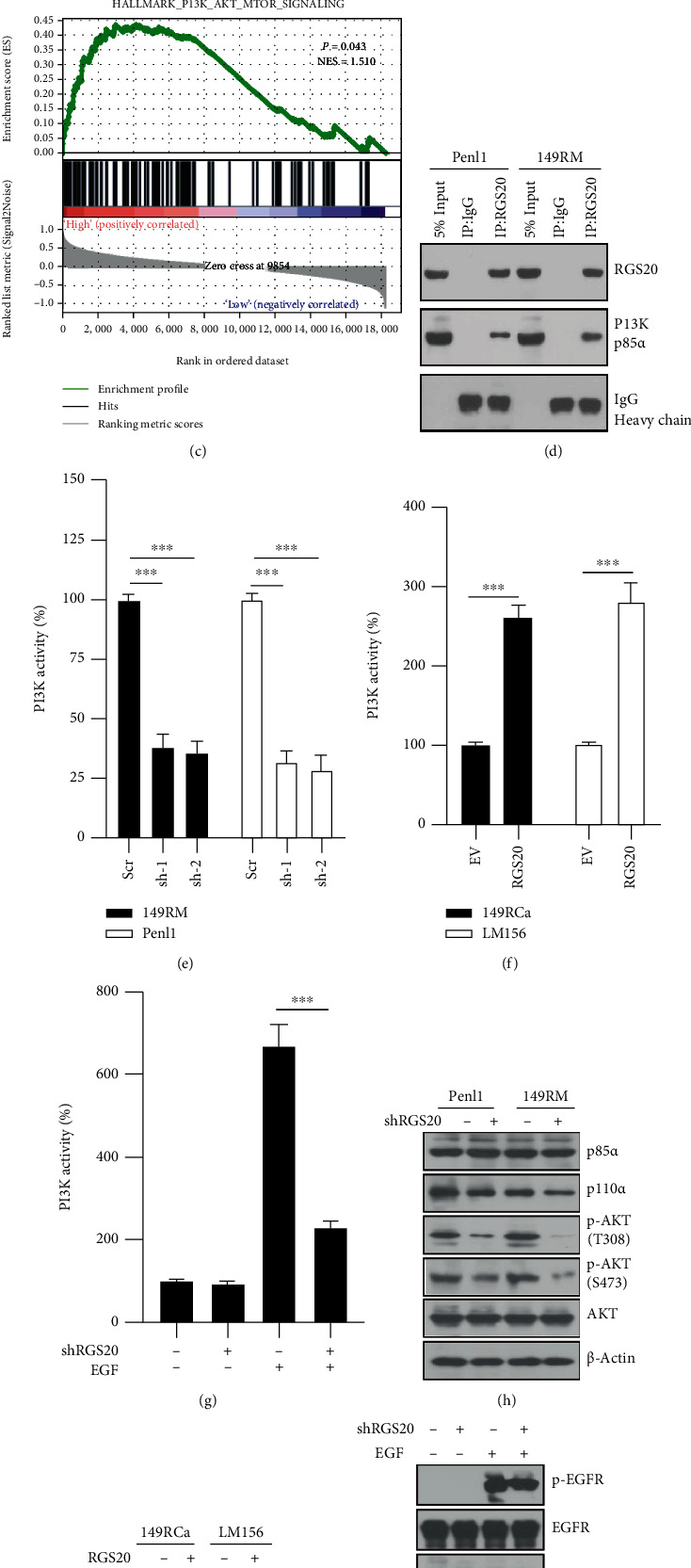
RGS20 regulates PI3K/AKT signaling activation in PC cells. **(**a) RNA-Seq analysis identified differentially expressed genes in Penl1 cells with or without RGS20 knockdown. (b) Top 5 enriched pathways for downregulated genes (*n* = 118) following RGS20 knockdown in Penl1 cells. (c) GSEA analysis revealed that RGS20-high PC cases exhibited highly enriched PI3K/AKT signaling in GSE57955 dataset. (d) Coimmunoprecitation analysis on RGS20 interaction with PI3K p85*α* subunit in PC cells. IgG was used as coimmunoprecitation control. (e) PI3K activity of RGS20-depleted 149RM and Penl1 cells. The PI3K activity in Scr control was regards as 100%. *n* = 4, ^∗∗∗^*P* < 0.001. (f) PI3K activity of 149RCa and LM156 cells with or without the RGS20 overexpression. The PI3K activity in EV control was regards as 100%. *n* = 4, ^∗∗∗^*P* < 0.001. (g) PI3K activity of RGS20-depleted Penl1 cells after EGF stimulation. After serum deprivation for 36 hours, Penl1 cells (Scr or shRGS20) were stimulated with EGF (50 ng/mL) for 30 min, and then PI3K activity was analyzed. *n* = 4, ^∗∗∗^*P* < 0.001. (h) Western blotting analysis on PI3K/AKT signaling proteins following RGS20 depletion in 149RM and Penl1 cells. (i) Western blotting analysis on PI3K/AKT signaling proteins following the overexpression of RGS20 in 149RCa and LM156 cells. (j) Western blotting analysis on PI3K/AKT signaling proteins in RGS20-depleted Penl1 cells after EGF stimulation. After serum deprivation for 36 hours, Penl1 cells (Scr or shRGS20) were stimulated with EGF (50 ng/mL) for 30 min. Western blotting was conducted to analyze PI3K/AKT signaling proteins.

RNA sequencing was conducted to identify the signaling pathways/genes impacted by RGS20 knockdown. As shown in Figure 4(a), the number of genes up- (log_2_FC ≥ 1, *P* < 0.05) or downregulated (log_2_FC ≤ −1, *P* < 0.05) in shRGS20 vs. Scr was 293 vs. 118, respectively (Supplementary Table S1). Pathway enrichment analysis of the downregulated genes (*n* = 118) using GO, KEGG, and Reactome Gene Set annotations showed that knockdown of RGS20 attenuated the activity of several signaling pathways, including the *PI3K/AKT signaling pathway*, *positive regulation of protein kinase activity*, and *positive regulation of response to an external stimulus* (Figure 4(b)). Additionally, GSEA analysis of the GSE57955 dataset showed that high RGS20 expression was consistent with significant enrichment of PI3K/AKT/mTOR signaling (Figure 4(c)). Some RGSs (RGS13, RGS16) are known to interact with PI3K p85*α* subunit and regulate PI3K activity [30, 31]. However, whether RGS20 could also serve as a potential interacting partner for PI3K p85*α* in PC still remains unknown. Coimmunoprecipitation analysis showed that RGS20 interacted with PI3K p85*α* in Penl1 and 149RM cells (Figure 4(d)). Therefore, these findings might connect the possible function of RGS20 with PI3K/AKT signaling activation in PC.

We next investigated the effect of RGS20 on PI3K activity in PC cells. Knockdown of RGS20 markedly attenuated PI3K activity in 149RM cells (Figure 4(e)). In contrast, PI3K activity was greatly increased in RGS20-expressing 149RCa and LM156 cells (Figure 4(f)). We also measured the PI3K activity following EGF stimulation in serum-deprived Penl1 cells with or without RGS20 depletion. Our results showed that PI3K activity was increased after the addition of EGF for 30 min, whereas knockdown of RGS20 greatly attenuated the EGF-induced PI3K activity (Figure 4(g)). The effect of RGS20 on PI3K/AKT signaling activation in PC cells was also examined by western blotting. Knockdown of RGS20 markedly attenuated PI3K downstream AKT phosphorylation (pS473/pT308) in 149RM and Penl1 cells, while the overexpression of RGS20 increased AKT phosphorylation (pS473/pT308) in 149RCa and LM156 cells (Figures 4(h) and 4(i)). Nevertheless, the western blotting analysis showed that knockdown of RGS20 markedly attenuated EGF-induced AKT phosphorylation (pS473/pT308) in Penl1 cells (Figure 4(j)).

### 3.5. Knockdown of PI3K p110*α* or p85*α* Attenuates Malignant Phenotypes in RGS20-High PC Cell Lines

**Figure 5:**
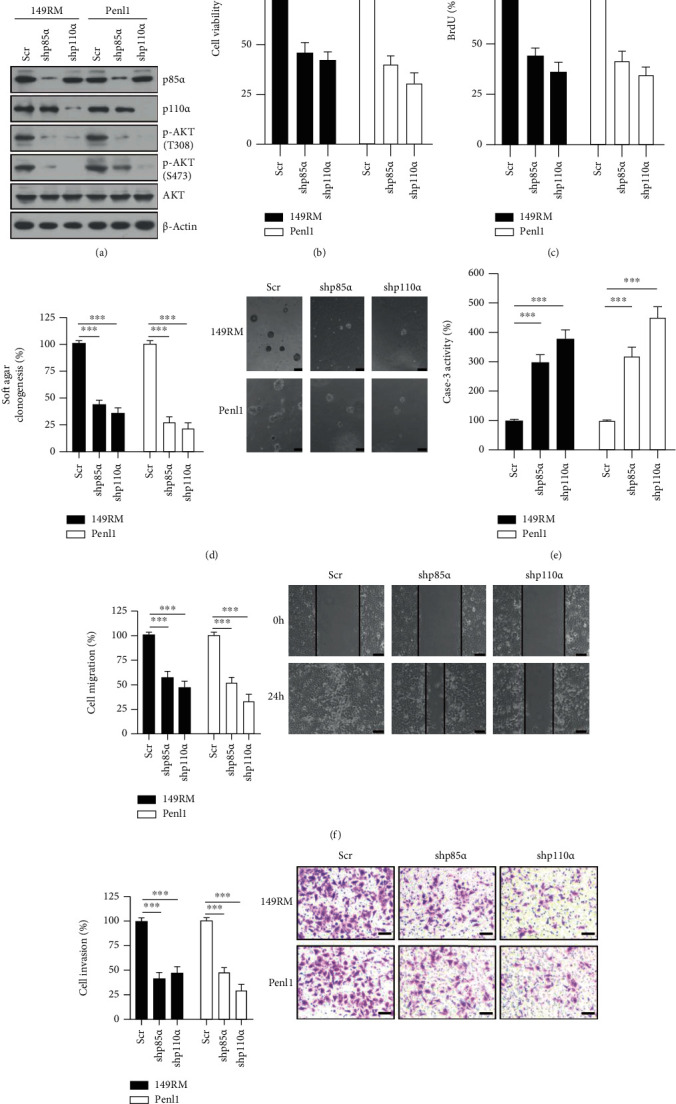
Knockdown of PI3K p85*α* or p110*α* suppresses cell proliferation, soft agar clonogenesis, and migration/invasion in PC cell lines. (a) Western blotting on PI3K p85*α* or p110*α* expression in 149RM and Penl1 cells transfected with scramble (Scr) control or shRNAs targeting p85*α* or p110*α*. *β*-Actin was used as loading control. (b) CCK-8 analysis on cell viability after p85*α* or p110*α* knockdown in 149RM and Penl1 cells. The cell viability in Scr control was regards as 100%. *n* = 4, ^∗∗∗^*P* < 0.001. (c) BrdU incorporation analysis on cell proliferation following p85*α* or p110*α* knockdown in 149RM and Penl1 cells. The BrdU incorporation in Scr control was regards as 100%. *n* = 4, ^∗∗∗^*P* < 0.001. (d) Soft agar clonogenesis of PC cells following p85*α* or p110*α* knockdown. The soft agar clonogenesis in Scr control was regards as 100%. *n* = 4, ^∗∗∗^*P* < 0.001. Bars: 100 *μ*m. (e) Caspase-3 activity of PC cells following p85*α* or p110*α* knockdown. The caspase-3 activity in Scr control was regards as 100%. *n* = 4, ^∗∗∗^*P* < 0.001. (f) Wound healing assay on PC cells following p85*α* or p110*α* knockdown. Micrographs showed the results of Penl1 cells. Bars: 100 *μ*m. The cell migration in Scr control was regards as 100%. *n* = 4, ^∗∗∗^*P* < 0.001. (g) Transwell invasion assay on PC cells following p85*α* or p110*α* knockdown. Bars: 50 *μ*m. The cell invasion in Scr control was regards as 100%. *n* = 4, ^∗∗∗^*P* < 0.001.

To evaluate the cellular function of PI3K signaling activation, we generated PI3K p85*α*/p110*α* knockdown cell models in PC cells. Knockdown of the PI3K p85*α*/p110*α* subunit greatly abolished AKT phosphorylation, reduced cell viability, and attenuated BrdU incorporation compared to that observed in the Scr control in 149RM and Penl1 cells (Figures 5(a)–5(c)). PI3K p85*α*/p110*α* knockdown also greatly impaired soft agar clonogenesis and increased caspase-3 activity compared to that observed in the Scr control in 149RM and Penl1 cells (Figures 5(d) and 5(e)). Furthermore, cell migration and invasion were significantly attenuated in shp85*α* or shp110*α* groups compared to Scr control in 149RM and Penl1 cells (Figures 5(f) and 5(g)).

### 3.6. The overexpression of Constitutively Activated PI3K p110*α* Restores Malignant Phenotypes in RGS20-Depleted PC Cells

**Figure 6:**
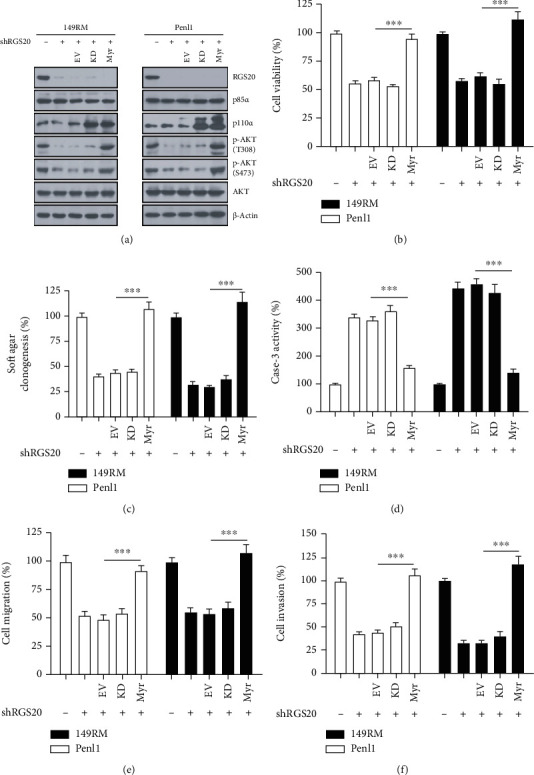
The overexpression of constitutively active PI3K p110*α* rescued malignant phenotypes impaired by RGS20 depletion. (a) Western blotting analysis on the expression of PI3K/AKT signaling pathway proteins after transfection with empty vector (EV), PI3K p110*α* Myr, or PI3K p110*α* KD plasmids in RGS20-depleted 149RM and Penl1cells. (b) CCK-8 analysis on cell viability after PI3K p110*α* Myr and KD plasmids transfection in RGS20-depleted 149RM and Penl1 cells. The cell viability in Scr control was regards as 100%. *n* = 4, ^∗∗∗^*P* < 0.001, as compared with EV. (c) Soft agar assay on clonogenesis after PI3K p110*α* Myr and KD plasmids transfection in RGS20-depleted 149RM and Penl1 cells. The soft agar clonogenesis in Scr control was regards as 100%. *n* = 4, ^∗∗∗^*P* < 0.001, as compared with EV. (d) Caspase-3 activity after PI3K p110*α* Myr and KD plasmid transfection in RGS20-depleted 149RM and Penl1 cells. The caspase-3 activity in Scr control was regards as 100%. *n* = 4, ^∗∗∗^*P* < 0.001, as compared with EV. (e) Wound healing assay on PC cells following PI3K p110*α* Myr and KD plasmid transfection in RGS20-depleted 149RM and Penl1 cells. Bars: 100 *μ*m. The cell migration in Scr control was regards as 100%. *n* = 4, ^∗∗∗^*P* < 0.001, as compared with EV. (f) Transwell invasion assay on PC cells following PI3K p110*α* Myr and KD plasmid transfection in RGS20-depleted 149RM and Penl1 cells. Bars: 50 *μ*m. The cell invasion in Scr control was regards as 100%. *n* = 4, ^∗∗∗^*P* < 0.001, as compared with EV.

The pCMV3 plasmids expressing PI3K p110*α* D933A (kinase dead, KD) or Myr (constitutively active) mutants were transfected into RGS20-depleted Penl1 and 149RM cells in order to confirm the hypothesis that PI3K/AKT signaling is required for RGS20 function. The overexpression of p110*α*/Myr mutant reactivated its downstream AKT signaling in RGS20-depleted Penl1 and 149RM cells (Figure 6(a)). The cellular function of PI3K p110*α* mutants was analyzed in Penl1 and 149RM cells. The overexpression of p110*α*/Myr mutant, but not p110*α*/KD, restored cell viability and soft agar clonogenesis attenuated by RGS20 knockdown in Penl1 and 149RM cells (Figures 6(b) and 6(c)). Moreover, caspase-3 activities induced by RGS20 knockdown were markedly reduced by the overexpression of p110*α*/Myr (Figure 6(d)). Further, compared to EV, the overexpression of p110*α*/Myr could markedly restored cell migration and invasion in RGS20-depleted Penl1 and 149RM cells (Figures 6(e) and 6(f)).

### 3.7. RGS20 Knockdown Disrupts PI3K/AKT Signaling and Suppresses Tumor Growth In Vivo

**Figure 7:**
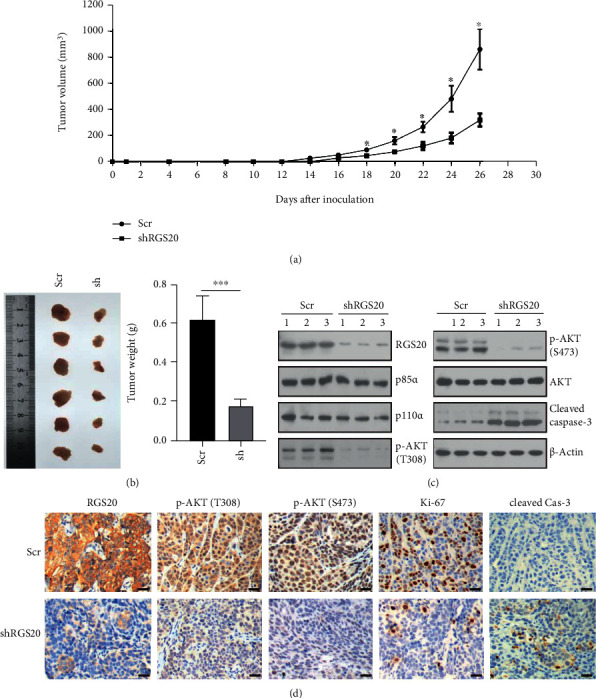
RGS20 knockdown suppressed in vivo tumor growth and disrupted PI3K/AKT signaling activation in the Penl1 xenograft model**. (**a). Nude mice xenograft study showed that RGS20 depletion attenuated subcutaneous tumor growth in nude mice. Tumor volume was measured every two days after Penl1 inoculation. *n* = 6, ^∗^*P* < 0.05, shRGS20 vs. Scr control. (b) Tumor weight of Penl1 xenografts with or without RGS20 knockdown. ^∗^*P* < 0.001. (c) Western blotting analysis on protein lysates extracted from Penl1 xenografts with or without RGS20 depletion (*n* = 3). (d) IHC staining on Penl1 xenografts with or without RGS20 depletion at day 26 after inoculation. The tissue sections were incubated with antibodies against indicated antibodies (RGS20, p-AKT (T308), p-AKT (S473), Ki-67, and cleaved caspase-3). Bar, 50 *μ*m.

The in vivo effect of RGS20 knockdown was evaluated in a Penl1 xenograft model. Penl1/shRGS20 tumors grew much slower than Penl1/Scr tumors (Figure 7(a)). As shown in Figure 7(b), knockdown of RGS20 reduced tumor weight compared to that observed in the Scr group. Additionally, RGS20 knockdown apparently reduced the level of p-AKT (pT308/pS473) and induced cleaved caspase-3 expression in Penl1 xenografts (Figure 7(c)). Furthermore, IHC staining showed that knockdown of RGS20 reduced the expression of p-AKT (pT308/pS473) and Ki-67 in Penl1 xenografts (Figure 7(d)). In contrast, the positivity of cleaved caspase-3 was greatly increased in RGS20-depleted Penl1 xenograft tissues (Figure 7(d)). Therefore, RGS20 might be crucial for PI3K/AKT signaling activation and tumor development in PC.

### 3.8. The aberrant RGS20 Expression Correlates with PI3K/AKT Signaling Activation in PC Specimens

**Figure 8:**
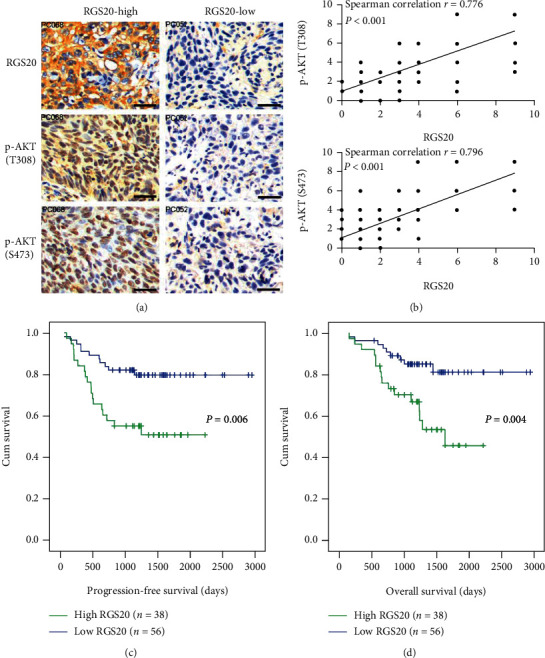
The high RGS20 expression is associated with PI3K/AKT signaling activation and unfavorable patient survival in PC. (a) IHC micrographs showed consistent expression of RGS20 and p-AKT (T308, S473) in RGS20-high or RGS20-low PCs. Bars: 100 *μ*m. (b) The RGS20 expression was significantly correlated with p-AKT (T308) and p-AKT (S473) in our PC cohort (*n* = 94). (c, d) Log-rank survival analysis showed that the high RGS20 expression was associated with progression-free survival and overall survival in our PC cohort (*n* = 94).

The expression of RGS20 and p-AKT (pT308/pS473) in clinical PC specimens was evaluated by IHC (*n* = 94). The expression of p-AKT (pT308/pS473) in PC cases with the high RGS20 expression was much higher than in those with the low RGS20 expression (Figures 8(a) and 8(b)). Additionally, survival analysis showed that the high RGS20 expression was significantly correlated with shorter progression-free survival and overall survival in our PC cohort (Figures 8(c) and 8(d)). Altogether, these data suggest that RGS20 is crucial for PI3K/AKT signaling activation in PC.

## 4. Discussion

RGS20 is an important regulator of neuronal G protein-coupled receptor signaling pathways in the brain [32]. Recently, RGS20 has been shown to play an important role in the carcinogenesis of breast, bladder, and renal cancer. In triple-negative breast cancer, the high RGS20 expression is correlated with nodal metastasis and poor clinical outcome [19]. In bladder cancer, the high RGS20 expression was associated with unfavorable clinical outcomes [20]. Additionally, the RGS20 expression has been shown to be correlated with immune cell infiltration in renal cancer [21]. However, the expression and function of RGS20 in PC remain unclear. Our analysis of the GSE57955 dataset revealed that RGS20 was highly expressed in PC compared to normal penile tissues. Consistently, IHC analysis showed that RGS20 was highly expressed in PC specimens compared to paired adjacent penile tissues, suggesting that RGS20 might serve as an oncogene in PC. Moreover, we showed that the high RGS20 expression was closely associated with higher tumor stage, nodal/pelvic metastasis, and unfavorable patient survival. These findings suggest that RGS20 might serve as a novel diagnostic and prognostic marker for PC.

The effect of RGS20 on the malignant phenotype (including uncontrollable cell proliferation, migration, and invasion) of cancer cells has been reported recently; although, the underlying mechanism remains largely unknown. Yang et al. [17] showed that RGS20 regulated cell aggregation and migration/invasion in several cancer cell lines, while Li et al. [20] showed that RGS20 promoted cell proliferation and migration in bladder cancer cells. In this study, a series of in vitro experiments were conducted to examine the effect of RGS20 on malignant phenotypes of PC cell line models. Our findings showed that knockdown of RGS20 attenuated cell proliferation, BrdU incorporation, clonogenesis, and migration/invasion in RGS20-high PC cell lines. In contrast, the overexpression of the RGS20 expression enhanced cell proliferation, BrdU incorporation, clonogenesis, and migration/invasion in RGS20-low PC cell lines. These findings suggest that RGS20 helps to maintain the malignant phenotype of PC. Thus, targeting RGS20 signaling might be useful to inhibit the malignant phenotype of PC.

PI3K/AKT signaling can promote cellular growth, survival, and disease dissemination in many cancers, and hyperactivation of PI3K/AKT signaling has been documented in PC [10, 33, 34]. However, the mechanism leading to PI3K/AKT signaling activation in PC remains poorly understood. In the present study, we showed that RGS20 could interact with PI3K p85*α* subunit and regulate PI3K/AKT signaling activation in PC, as manipulation of the RGS20 expression affected PI3K activity and downstream AKT phosphorylation. In addition, knockdown of PI3K p85*α*/p110*α* subunits attenuated cell proliferation, BrdU incorporation, clonogenesis, and migration/invasion in PC cell lines. Moreover, we established the functional association between RGS20-regulated PI3K/AKT pathway activation and malignant progression of PC, as the overexpression of constitutively activated PI3K p110*α*, rather than its kinase-dead mutant, rescued cell proliferation, and cell migration/invasion attenuated by RGS20 knockdown in PC cells. Therefore, the high RGS20 expression might activate the PI3K/AKT pathway to maintain the malignant phenotype of PC. Recently, Li et al. showed that RGS20 could activate the downstream NF-*κ*B signaling pathway in bladder cancer [20]. These findings suggested that RGS20 might exert multifaceted functions in regulating oncogenic signaling (PI3K/AKT, NF-*κ*B) in different cancers.

The oncogenic function of RGS20 was confirmed in in vivo xenograft studies. Consistent with the in vitro findings, RGS20 depletion suppressed transplanted tumor growth, attenuated PI3K/AKT signaling, and induced apoptosis in vivo. Furthermore, GSEA and IHC analysis revealed the correlation between high RGS20 expression and PI3K/AKT signaling activation in clinical PC specimens, confirming the clinical relevance of RGS20/PI3K/AKT signaling in PC. Therefore, targeting RGS20 might be a useful strategy to abolish PI3K/AKT signaling activation and to suppress tumor development in PC.

In conclusion, it was found that the high RGS20 expression could serve as a potential diagnostic and prognostic biomarker for PC, and RGS20 might regulate PI3K/AKT signaling activation to promote PC progression. Our findings highlighted the essential role of RGS20 in modulating the PI3K/AKT signaling activity in PC, which may help to develop RGS20-targeting therapeutics in the future.

## Figures and Tables

**Table 1:** Association between clinicopathologic characteristics and RGS20 expression in PC cohort.

Clinicopathological parameters	RGS20 low expression	RGS20 high expression	*P* value
Age (year)			0.502
≤53	27	21	
>53	29	17	
Phimosis			0.106
Yes	39	32	
No	17	6	
Body mass index			0.347
<24	41	31	
≥24	15	7	
Pathological grade			0.379
G1	43	32	
G2 + G3	13	6	
Histological subtype			0.101
Usual	32	28	
Others	24	10	
T stage			0.028
Ta + T1	28	11	
T2 + T3	28	27	
Nodal status			0.002
Negative	40	15	
Positive	16	23	
Pelvic metastasis			0.013
No	56	34	
Yes	0	4	

## Data Availability

The original contributions presented in the study are included in the article/supplementary material; further inquiries can be directed to the corresponding authors.

## Supplementary Materials

Supplementary Materials Table S1: differentially expressed genes in shRGS20 vs. Scr comparison in Penl1.
