# Supplementary material for: RGS20 Promotes Tumor Progression through Modulating PI3K/AKT Signaling Activation in Penile Cancer
Source: J Oncol. 2022 Apr 19;2022:1293622. doi: 10.1155/2022/1293622 (PMC9042636; doi:10.1155/2022/1293622)
Supplement: Supplementary Materials — Table S1: differentially expressed genes in shRGS20 vs. Scr comparison in Penl1. [file 1293622.f1.pdf]

Supplementary methods

Generation of lentivirus particles

Lentiviruses encoding shRNAs and control scramble were purchased from Genecopoeia Inc. Lentiviral particles were produced by transient cotransfection of packaging plasmids into the Lenti-Pac 293Ta cell line. Produced lentiviruses were concentrated by using Centricon Plus-20 centrifugal filter device (Millipore). The oligo sequences for shRNAs are shown as follows:

Scramble shRNA: CCTAAGGTTAAGTCGCCCTCG

shRNA for RGS20 sh-1: CATGAACTCTGCTGTCTATAA

shRNA for RGS20 sh-2: CATCTTCTGCTGGAGTAATAC

shRNA for PI3K p85α: GCGGTACAGCAAAGAATACAT

shRNA for PI3K p110α: GCATTAGAATTTACAGCAAGA

Bromodeoxyuridine (BrdU) incorporation assay

BrdU incorporation was measured by a BrdU cell proliferation assay kit (Cell Signaling, Boston, MA). Briefly, PC cells were plated (2×10^3 cells /well) into 96-well plate and incubated for 96 hours. After incubation, cells were labeled with 10 μM BrdU for 2 hours, then fixed and the DNA was denatured with fixing/denaturing solution. BrdU mouse antibody was added to detect the incorporated BrdU. The absorbance (OD_{450}) was measured using a Multiskan MK3 microplate reader (Thermo Fisher Scientific, Waltham, MA, USA).

Wound healing assay

Wound healing assay was used to evaluate the cell migratory ability. Briefly, PC cells (5×10^5) were grown in six-well plates for 48 h until the cells grew confluent. A sterile 200 μl pipette tip was used to make a scratch wounds in the center of the well. After indicated time, the distance between the wound sides was measured.

Transwell invasion assay

Cell invasion assay were performed based on transwell chamber with 8 μm pores (Corning). PC cells (5 × 10^5 cells/well) were seeded in transwell inserts pre-coated with 50 μl Matrigel. The plates were incubated for 36 h at 37 °C. The invaded cells on the bottom surface of the membrane were fixed by dehydrated alcohol, and stained by 0.2% crystal violet solution (Sigma-Aldrich, USA). After wash with deionized water, Penl1 cells were photographed with Olympus BX43 microscope. The crystal violet in stained cells were eluted by 20% glacial acetic acid and measured with a Thermo MK3 microplate reader at 570 nm.

Table S1: Differentially expressed genes in shRGS20 vs. Scr comparison in Penl1

AccID	Penl1 Scr1	Penl1 scr2	Penl1 scr3	Penl1 KD1	Penl1 KD2	Penl1 KD3	average	log2FC	P value	log10pvalue
MIR3192	0.263	0.409	0.138	0.000	0.000	0.071	0.087	-3.515	0.039	1.404
TEX19	0.198	0.175	0.207	0.000	0.069	0.000	0.119	-3.074	0.002	2.628
RPL11P3	0.329	0.818	0.965	0.000	0.000	0.284	0.134	-2.897	0.047	1.332
RN7SKP27	1.251	0.701	0.483	0.061	0.275	0.000	0.138	-2.855	0.045	1.344
FLJ22447	0.395	0.292	0.207	0.061	0.069	0.000	0.145	-2.782	0.012	1.913
PKD2L2	0.395	0.234	0.276	0.061	0.000	0.071	0.146	-2.776	0.008	2.076
CYP1D1P	0.198	0.117	0.138	0.000	0.069	0.000	0.152	-2.716	0.019	1.732
EIF4A1P7	0.659	0.993	0.414	0.000	0.207	0.142	0.169	-2.568	0.033	1.484
SLC2A1-AS	0.527	0.234	0.483	0.000	0.069	0.142	0.169	-2.561	0.026	1.581
IL7R	4.215	4.381	4.895	1.162	0.482	0.709	0.174	-2.520	0.000	3.692
RPL10P12	0.198	0.350	0.207	0.061	0.000	0.071	0.175	-2.516	0.019	1.729
RPS15AP2	0.198	0.409	0.552	0.000	0.138	0.071	0.180	-2.473	0.045	1.343
IGKV1OR2	0.263	0.175	0.276	0.061	0.069	0.000	0.182	-2.459	0.007	2.147
ATF4P3	0.132	0.117	0.069	0.061	0.000	0.000	0.193	-2.377	0.037	1.429
RPS15AP3	0.263	0.117	0.276	0.061	0.000	0.071	0.201	-2.313	0.035	1.457
KLHL7-AS	0.922	0.467	0.896	0.183	0.069	0.213	0.203	-2.298	0.017	1.772
PTGES3P3	0.395	0.350	0.276	0.000	0.069	0.142	0.206	-2.278	0.007	2.134
RPS20P33	0.395	0.409	0.483	0.061	0.138	0.071	0.210	-2.254	0.001	3.137
PITRM1-AS	0.527	1.051	0.620	0.183	0.069	0.213	0.211	-2.242	0.026	1.585
SOS2	39.977	40.071	37.919	9.249	7.722	8.349	0.215	-2.220	0.000	5.506
KCNE3	0.856	0.526	1.241	0.245	0.275	0.071	0.225	-2.150	0.035	1.455
ATP6V0D2	0.329	0.876	0.758	0.245	0.069	0.142	0.232	-2.109	0.044	1.352
TM4SF1-AS	0.856	0.701	0.689	0.122	0.344	0.071	0.239	-2.064	0.005	2.335
SNORA11F	0.988	0.467	0.758	0.183	0.275	0.071	0.239	-2.063	0.026	1.592
RASGRF2	0.263	0.350	0.414	0.183	0.069	0.000	0.245	-2.026	0.020	1.699
SNORA12	1.185	0.701	0.965	0.000	0.207	0.496	0.246	-2.021	0.023	1.630
SNORA12	1.185	0.701	0.965	0.000	0.207	0.496	0.246	-2.021	0.023	1.630
SNORA12	1.185	0.701	0.965	0.000	0.207	0.496	0.246	-2.021	0.023	1.630
U2AF1L4	0.527	1.168	0.965	0.183	0.207	0.284	0.253	-1.982	0.026	1.586
PTGER1	0.659	0.409	0.689	0.183	0.000	0.284	0.266	-1.912	0.024	1.618
AREG	17.255	16.356	22.890	4.585	5.920	4.678	0.269	-1.896	0.003	2.562
HMGA1P8	0.724	0.526	0.689	0.183	0.069	0.284	0.276	-1.856	0.006	2.235
FGFR1	1.581	0.701	1.103	0.489	0.239	0.213	0.278	-1.847	0.039	1.410
MINDY4	0.263	0.467	0.414	0.183	0.069	0.071	0.282	-1.824	0.019	1.724
EREG	93.983	83.589	82.457	21.764	22.853	29.701	0.286	-1.807	0.000	3.818
PTGES2-AS	1.317	0.876	0.827	0.306	0.207	0.354	0.287	-1.801	0.011	1.945
BCAT1	7.640	7.068	8.135	1.345	2.891	2.339	0.288	-1.797	0.001	3.234
TTBK1	0.132	0.175	0.138	0.061	0.069	0.000	0.292	-1.775	0.015	1.824
PDGFA	17.058	16.239	12.962	5.559	4.673	3.357	0.294	-1.767	0.001	2.825
PJVK	0.395	0.584	0.414	0.061	0.207	0.142	0.294	-1.767	0.011	1.955
SLFNL1-AS	0.395	0.759	0.620	0.122	0.069	0.354	0.307	-1.702	0.041	1.389
ACMSD	1.515	1.051	0.689	0.245	0.551	0.213	0.310	-1.692	0.046	1.338
RGS1	4.940	4.381	7.584	1.039	1.927	2.268	0.310	-1.691	0.021	1.678
ASNS	1.976	2.278	2.137	0.856	0.620	0.567	0.320	-1.646	0.000	3.506
SLC2A5	0.724	0.643	0.483	0.245	0.069	0.284	0.323	-1.632	0.013	1.900
PSAT1	198.965	202.693	203.593	63.520	62.778	70.035	0.324	-1.624	0.000	6.032
FAM129A	3.622	3.446	5.584	1.406	2.065	0.780	0.336	-1.574	0.023	1.641
C11orf44	0.856	0.701	0.827	0.183	0.620	0.000	0.337	-1.570	0.050	1.301
OR7E12P	0.329	0.292	0.345	0.122	0.207	0.000	0.340	-1.555	0.027	1.576
INHBE	1.910	2.278	2.413	1.100	0.757	0.425	0.346	-1.532	0.004	2.370
SPX	7.706	8.003	7.515	2.201	2.272	3.615	0.348	-1.522	0.000	3.329
SLCO4A1-	1.185	1.110	1.517	0.428	0.688	0.213	0.349	-1.520	0.011	1.950
CCND3	58.879	59.815	63.842	23.040	17.309	23.311	0.349	-1.520	0.000	4.048
ALDH1L2	23.512	27.220	26.061	7.397	10.257	9.144	0.349	-1.519	0.000	3.575
CCNE2	11.921	14.194	11.445	3.559	4.949	4.648	0.350	-1.514	0.001	2.995
EFNA5	23.380	24.066	24.475	6.114	9.962	9.210	0.352	-1.508	0.000	3.660
MTG1	1.712	1.752	2.344	0.306	0.551	1.205	0.355	-1.495	0.021	1.682
ZMAT4	1.515	1.227	1.103	0.183	0.551	0.638	0.357	-1.486	0.011	1.950
PPIAP31	1.712	1.519	1.448	0.428	0.964	0.284	0.358	-1.482	0.011	1.973
TAS2R20	1.712	2.804	2.137	0.978	0.413	0.992	0.358	-1.481	0.018	1.734
TOMM20L	0.593	0.643	0.689	0.061	0.275	0.354	0.359	-1.478	0.011	1.957
TNNI1	1.778	2.161	1.241	1.100	0.207	0.567	0.362	-1.467	0.042	1.381
FAM35BP	0.659	0.584	0.827	0.061	0.344	0.354	0.367	-1.446	0.022	1.658
E2F8	16.729	18.400	16.753	5.315	6.949	6.932	0.370	-1.435	0.000	3.830
CLDN20	0.461	0.350	0.483	0.061	0.207	0.213	0.371	-1.430	0.013	1.871
ANGPTL1	0.593	0.584	0.483	0.061	0.344	0.213	0.372	-1.425	0.018	1.755
RHOXF1-AS	1.317	0.876	0.689	0.306	0.207	0.567	0.374	-1.417	0.049	1.310
KRBA2	0.329	0.409	0.483	0.183	0.138	0.142	0.379	-1.399	0.006	2.250
STC2	49.395	48.424	48.813	18.952	18.241	18.997	0.383	-1.384	0.000	6.850
PTGS2	96.354	99.594	98.728	37.171	38.066	37.994	0.384	-1.380	0.000	6.331
NPR3	16.992	20.036	16.891	6.175	8.123	6.521	0.386	-1.373	0.001	3.119
HMCN1	20.614	14.311	13.030	6.664	7.021	5.033	0.390	-1.357	0.016	1.801
MYC	157.999	168.697	173.947	71.792	60.043	63.816	0.391	-1.355	0.000	4.197
GTF2IP7	12.316	13.493	18.615	4.585	6.952	5.883	0.392	-1.351	0.012	1.928
MCF2L2	1.647	2.453	1.930	0.856	1.033	0.496	0.395	-1.338	0.013	1.890
AFAP1-AS	3.820	2.453	3.654	1.162	1.377	1.418	0.398	-1.327	0.010	1.981
ZNF710-AS	1.844	1.343	1.793	0.795	0.344	0.851	0.400	-1.324	0.012	1.937
COL6A3	45.510	43.810	43.090	17.546	14.937	20.911	0.403	-1.310	0.000	3.830
KCTD9P2	0.659	0.467	0.552	0.122	0.138	0.425	0.409	-1.292	0.043	1.367
FEM1A	1.054	1.519	0.965	0.489	0.757	0.213	0.412	-1.278	0.041	1.389
ITPKA	22.985	27.104	25.303	11.555	10.325	9.357	0.414	-1.271	0.000	3.395
RPL4P5	1.581	1.285	1.103	0.245	0.413	0.992	0.416	-1.266	0.044	1.359

CXCL17	3.030	3.271	4.481	1.712	1.652	1.134	0.417	-1.261	0.012	1.904
VEGFA	128.165	143.696	152.298	69.643	59.017	48.525	0.418	-1.259	0.001	3.041
RNU6-26P	1.515	2.687	2.137	0.550	0.688	1.418	0.419	-1.255	0.047	1.328
STARD4	35.631	37.209	31.370	15.345	14.731	14.461	0.427	-1.226	0.000	3.453
ADM2	4.874	6.017	4.275	1.467	2.891	2.127	0.428	-1.226	0.012	1.936
OLFM2	1.976	1.928	1.034	0.795	0.482	0.851	0.431	-1.215	0.046	1.340
ZNF773	0.461	0.759	0.620	0.306	0.207	0.284	0.432	-1.210	0.019	1.725
FAM86B1	1.910	1.986	2.689	1.039	0.964	0.851	0.433	-1.206	0.008	2.094
FGF2	15.807	15.772	13.168	7.336	5.463	6.640	0.434	-1.203	0.001	2.916
KCNE4	36.750	35.048	36.816	16.384	15.006	16.091	0.437	-1.194	0.000	5.046
ASB4	5.598	5.666	6.205	1.956	2.409	3.332	0.441	-1.182	0.002	2.722
IL20RB	4.149	5.958	5.584	1.712	2.478	2.765	0.443	-1.174	0.010	1.995
PHGDH	55.191	57.887	61.085	27.695	25.676	24.739	0.448	-1.157	0.000	4.126
CPHL1P	6.323	7.535	6.757	2.507	2.960	3.828	0.451	-1.149	0.002	2.701
TRPM8	0.461	0.701	0.620	0.245	0.138	0.425	0.453	-1.142	0.041	1.383
TM4SF4	6.323	4.264	3.654	2.690	2.134	1.630	0.453	-1.142	0.040	1.401
SLC1A2	3.030	4.030	3.378	1.834	1.377	1.559	0.457	-1.130	0.004	2.375
JAZF1	10.143	7.944	9.721	4.524	4.956	3.332	0.461	-1.118	0.004	2.416
CDKL3	0.659	0.584	0.620	0.367	0.138	0.354	0.461	-1.117	0.012	1.906
TM4SF19	7.574	6.250	7.860	3.301	2.960	3.757	0.462	-1.114	0.002	2.685
CNKSR2	7.640	9.521	10.893	4.585	4.061	4.324	0.462	-1.113	0.006	2.205
VSIG1	295.384	298.257	287.084	135.416	138.842	139.502	0.470	-1.090	0.000	5.776
LAD1	75.542	69.979	68.117	38.088	33.867	29.772	0.476	-1.070	0.000	3.469
RPP21	0.724	0.876	1.034	0.489	0.275	0.496	0.478	-1.064	0.016	1.785
CLSPN	31.218	25.118	26.888	14.306	14.662	11.058	0.481	-1.056	0.003	2.592
TUBA1A	1.383	2.103	1.517	0.734	0.826	0.851	0.482	-1.054	0.018	1.741
NECAB1	6.191	6.542	6.412	2.507	3.511	3.261	0.485	-1.045	0.000	3.303
FAM156A	0.527	0.818	0.827	0.428	0.413	0.213	0.485	-1.044	0.037	1.438
C6orf223	3.227	4.439	4.688	2.079	1.652	2.268	0.486	-1.042	0.012	1.916
FBXO36	7.772	5.199	6.825	3.729	2.822	3.119	0.489	-1.034	0.013	1.875
SCG2	1.581	1.227	1.310	0.489	0.964	0.567	0.491	-1.027	0.018	1.737
CDCP1	4.215	5.432	4.412	2.445	1.859	2.623	0.493	-1.021	0.006	2.238
POLR3G	12.513	14.603	12.548	6.970	7.159	5.458	0.494	-1.018	0.002	2.803
CALCRL	0.527	0.759	0.896	0.245	0.413	0.425	0.496	-1.011	0.040	1.394
MILR1	12.843	13.844	14.271	6.908	6.746	6.734	0.498	-1.006	0.000	4.055
SLC38A5	26.015	24.826	27.922	13.083	12.941	13.326	0.500	-1.001	0.000	3.875
TRIM31	42.546	44.978	43.711	83.512	94.374	85.062	2.004	1.003	0.000	3.651
TBC1D3L	1.976	1.227	1.448	3.668	2.616	3.048	2.007	1.005	0.014	1.839
PIK3CD	1.647	2.453	1.999	3.240	4.474	4.537	2.009	1.006	0.013	1.881
METTL27	2.832	4.381	2.620	7.031	6.746	6.025	2.014	1.010	0.006	2.207
IL3RA	0.198	0.701	0.758	1.100	0.964	1.276	2.016	1.011	0.048	1.315
KIF20A	97.013	94.162	101.486	198.876	197.352	194.580	2.019	1.013	0.000	5.639
UBALD2	50.054	57.070	48.813	107.966	107.590	99.806	2.022	1.016	0.000	3.865
C1QTNF1	19.626	19.276	22.131	42.000	40.200	41.255	2.023	1.016	0.000	4.434
CYP2S1	63.555	70.329	77.976	148.866	142.215	138.014	2.025	1.018	0.000	3.803
BAIAP3	8.694	6.192	8.204	15.529	14.593	16.658	2.026	1.019	0.001	2.906
KRT80	9.352	8.996	8.756	17.240	19.205	18.572	2.030	1.021	0.000	3.984
GDPD3	0.724	0.993	0.827	1.528	2.134	1.559	2.052	1.037	0.014	1.868
COQ8A	19.824	19.685	21.166	44.079	37.860	42.744	2.055	1.039	0.000	3.403
TRAV19	0.263	0.467	0.758	1.100	0.826	1.134	2.055	1.039	0.039	1.406
C4orf36	2.042	1.869	2.206	4.341	4.337	3.899	2.056	1.040	0.000	3.591
AASS	2.371	1.402	2.413	3.974	3.648	5.175	2.069	1.049	0.018	1.744
PI15	0.856	1.227	0.483	1.773	1.859	1.701	2.079	1.056	0.014	1.864
VASH1	1.317	1.285	1.310	3.546	2.685	1.914	2.082	1.058	0.040	1.395
RGS11	1.054	1.168	0.965	1.956	2.340	2.339	2.082	1.058	0.001	2.913
HCG15	0.461	0.234	0.483	0.856	0.826	0.780	2.091	1.064	0.007	2.180
ZSCAN12P1	0.593	0.818	0.689	1.406	1.721	1.276	2.097	1.068	0.006	2.190
MAP1LC3A	8.496	9.521	7.170	17.974	17.828	17.012	2.097	1.068	0.000	3.612
NT5M	0.922	1.343	0.896	2.323	2.203	2.127	2.104	1.073	0.002	2.764
PDK4	6.454	7.360	6.067	13.694	13.561	14.602	2.105	1.074	0.000	3.886
BDNF-AS	0.461	0.350	0.276	0.611	0.757	0.922	2.106	1.075	0.019	1.732
NDRG2	2.964	3.096	2.620	6.358	5.989	5.954	2.109	1.076	0.000	4.124
TINAGL1	21.207	23.132	27.440	56.306	51.283	44.020	2.112	1.079	0.003	2.571
CDC42BPG	1.712	1.051	1.310	2.690	3.511	2.410	2.114	1.080	0.017	1.777
AMH	1.317	0.643	1.241	1.895	2.822	2.056	2.116	1.081	0.029	1.539
CPNE5	0.922	0.467	0.827	1.773	1.377	1.559	2.124	1.087	0.010	2.006
DAPK2	8.694	11.741	10.135	20.664	23.817	20.486	2.125	1.088	0.001	2.923
DNAH1	13.501	11.332	12.824	27.022	28.085	25.093	2.130	1.091	0.000	3.704
SYNPO	6.388	3.855	6.894	10.943	12.941	12.759	2.138	1.096	0.005	2.336
CAPN10-AS	1.185	1.168	0.414	2.017	2.272	1.630	2.139	1.097	0.029	1.536
OSER1-AS1	1.647	1.752	2.413	3.852	3.924	4.678	2.143	1.099	0.003	2.464
PKD1P6	6.718	7.360	6.274	16.384	12.803	14.531	2.148	1.103	0.002	2.706
RGL3	1.120	1.694	0.689	2.201	2.340	3.048	2.167	1.115	0.025	1.595
LRRC75B	2.108	2.862	0.689	3.974	4.543	3.757	2.169	1.117	0.031	1.503
CRTC1	6.652	5.316	4.619	14.550	10.945	10.633	2.178	1.123	0.009	2.025
PDK2	5.862	5.549	5.653	13.572	13.974	9.640	2.179	1.124	0.008	2.076
SOST	0.132	0.292	0.483	0.795	0.620	0.567	2.186	1.128	0.043	1.366
AQP3	13.897	13.610	14.547	32.402	30.081	29.488	2.187	1.129	0.000	4.239
MXD4	15.016	17.290	12.548	30.201	34.969	33.033	2.189	1.131	0.001	3.098
IRS2	6.323	7.535	7.308	14.795	14.456	17.154	2.192	1.133	0.001	3.088
TAS2R4	0.329	0.292	0.345	0.795	0.688	0.638	2.196	1.135	0.001	2.856
ETV7	0.132	0.292	0.207	0.489	0.482	0.425	2.214	1.147	0.007	2.141
IL37	0.132	0.117	0.138	0.367	0.207	0.284	2.217	1.149	0.028	1.547
RCBTB2	1.383	2.103	2.275	3.668	4.887	4.253	2.223	1.153	0.006	2.207

VASN	4.610	5.024	2.551	8.804	8.191	10.137	2.227	1.155	0.006	2.189
C1QL1	1.317	0.584	0.689	2.507	1.583	1.701	2.235	1.160	0.045	1.350
ACTL8	1.712	3.154	2.551	5.441	4.887	6.309	2.243	1.165	0.006	2.194
TAGLN	1.515	0.759	1.517	2.751	2.960	2.835	2.254	1.173	0.004	2.441
SCNN1A	12.645	12.968	14.754	30.629	29.531	31.402	2.268	1.182	0.000	4.437
SMG1P4	3.227	4.089	4.688	6.970	9.981	10.278	2.268	1.182	0.011	1.951
LBX2	1.383	1.227	1.103	2.507	3.511	2.410	2.270	1.183	0.012	1.916
TPM3P6	0.132	0.175	0.276	0.489	0.413	0.425	2.278	1.188	0.007	2.154
C3	1.185	1.285	1.172	2.201	2.340	3.757	2.278	1.188	0.036	1.447
RNPS1P1	0.066	0.117	0.138	0.245	0.207	0.284	2.291	1.196	0.011	1.957
NR6A1	2.437	1.928	1.586	4.280	4.956	4.466	2.303	1.203	0.001	2.898
KLRC2	3.425	2.512	3.654	7.336	5.851	8.932	2.306	1.206	0.012	1.922
GPR176	1.515	2.337	3.102	6.358	5.025	4.678	2.310	1.208	0.012	1.938
OR2I1P	9.418	9.171	10.824	20.909	26.571	20.557	2.313	1.210	0.003	2.511
SPINK1	22.129	24.709	22.614	49.153	54.931	56.850	2.317	1.212	0.000	3.624
SOX18	1.185	1.460	2.758	5.563	3.235	3.757	2.324	1.216	0.050	1.305
MREG	0.263	0.350	0.207	0.795	0.620	0.496	2.328	1.219	0.019	1.710
ACBD7	0.856	1.752	1.448	2.873	3.098	3.473	2.328	1.219	0.005	2.325
NPIPB2	2.371	2.220	1.034	4.646	3.992	4.608	2.355	1.236	0.006	2.237
ERBB3	64.807	55.843	61.498	144.587	139.048	148.930	2.375	1.248	0.000	4.561
AGBL1	0.724	0.526	0.483	1.528	1.101	1.489	2.377	1.249	0.007	2.163
RPL12P14	1.778	0.818	1.379	3.424	2.891	3.190	2.391	1.258	0.004	2.354
DEFB131B	0.461	0.409	0.207	0.917	1.101	0.567	2.401	1.264	0.045	1.345
HNRNPA3P	0.395	0.234	0.069	0.489	0.620	0.567	2.402	1.264	0.033	1.487
GLIPR2	1.515	0.701	1.034	2.507	2.547	2.765	2.406	1.266	0.004	2.438
RAB37	0.922	2.395	3.240	5.625	4.130	6.025	2.406	1.267	0.026	1.586
FBXO16	0.066	0.292	0.138	0.428	0.344	0.425	2.415	1.272	0.032	1.500
NPIPA5	1.449	1.694	1.930	3.301	5.094	3.899	2.423	1.277	0.012	1.938
TMEM191A	0.922	0.584	0.552	1.651	2.134	1.205	2.425	1.278	0.029	1.537
ATOH8	11.789	11.624	16.202	33.564	32.284	30.268	2.426	1.279	0.000	3.345
EDARADD	0.395	0.526	0.000	0.795	0.757	0.709	2.455	1.296	0.049	1.308
CECR2	0.198	0.467	0.758	1.345	0.826	1.347	2.472	1.305	0.042	1.374
MEGF6	16.333	19.685	20.408	50.682	43.848	45.012	2.473	1.306	0.000	3.454
UNC13A	0.988	1.460	1.172	3.057	2.960	3.119	2.523	1.335	0.000	3.651
SPNS2	0.724	1.051	0.483	2.017	1.927	1.772	2.531	1.340	0.003	2.518
RCN3	2.503	2.921	1.930	6.542	6.815	5.316	2.539	1.344	0.002	2.648
RORC	2.042	1.110	0.896	4.402	2.616	3.261	2.539	1.344	0.030	1.523
PTPRN2	0.922	0.935	0.345	2.262	1.790	1.559	2.549	1.350	0.016	1.793
TMEM212-	0.395	0.234	0.138	0.489	0.688	0.780	2.553	1.352	0.025	1.596
CTSK	2.503	2.395	0.896	5.319	4.405	5.104	2.559	1.356	0.007	2.165
AZIN2	0.659	0.526	0.138	1.162	1.308	0.922	2.565	1.359	0.023	1.635
SESN3	0.593	0.876	1.034	2.017	2.134	2.268	2.565	1.359	0.001	3.039
PRAF2	0.066	0.818	0.483	1.345	1.239	0.922	2.566	1.359	0.047	1.325
UBBP4	0.461	0.993	0.276	1.162	1.790	1.489	2.567	1.360	0.033	1.488
PIK3IP1	3.030	4.615	2.344	8.987	7.641	9.073	2.573	1.364	0.003	2.517
MRAS	0.790	0.175	0.276	1.039	1.033	1.134	2.583	1.369	0.028	1.559
TXK	1.251	1.636	2.275	2.935	4.405	6.025	2.589	1.372	0.044	1.358
EFCAB12	0.461	0.526	1.103	1.467	2.203	1.772	2.604	1.381	0.019	1.713
PAPLN	5.993	5.316	3.034	10.882	15.075	11.413	2.606	1.382	0.009	2.069
ZNF219	1.054	1.343	0.896	3.301	2.478	2.835	2.616	1.387	0.003	2.545
FAM167B	0.132	0.175	0.000	0.245	0.275	0.284	2.617	1.388	0.037	1.426
CCDC148	0.659	1.285	0.207	1.528	1.927	2.197	2.629	1.394	0.034	1.471
NR3C2	0.856	1.051	0.965	1.528	3.373	2.694	2.644	1.403	0.044	1.358
AZGP1	3.359	2.453	3.034	6.542	9.844	7.018	2.646	1.404	0.010	1.986
ASPDH	0.395	0.234	0.207	0.550	1.033	0.638	2.657	1.410	0.044	1.353
ROBO3	1.515	0.876	0.414	1.651	2.685	3.119	2.658	1.410	0.045	1.342
NEIL1	0.527	0.350	0.965	1.590	1.927	1.418	2.678	1.421	0.012	1.920
PSORS1C3	0.724	0.701	0.620	1.651	1.652	2.197	2.688	1.427	0.003	2.472
HIST1H2A	7.113	6.834	7.998	18.585	19.136	21.549	2.701	1.433	0.000	3.662
VKORC1	0.066	0.117	0.207	0.428	0.344	0.284	2.710	1.438	0.019	1.711
KRT7	5.862	6.776	7.170	17.240	19.894	16.587	2.712	1.439	0.000	3.323
YPEL3	8.628	8.295	8.549	25.555	23.886	20.628	2.751	1.460	0.001	3.292
PKD1P5	0.988	0.350	0.965	2.201	1.514	2.694	2.782	1.476	0.027	1.570
KLF2	2.503	3.154	4.275	9.109	10.807	7.797	2.790	1.481	0.004	2.371
CYP26B1	4.610	4.498	3.723	11.738	12.253	11.838	2.792	1.481	0.000	4.743
TBC1D3D	0.988	0.759	0.207	1.834	2.409	1.276	2.824	1.498	0.041	1.383
TXNIP	14.884	13.493	13.030	37.232	41.026	39.129	2.835	1.503	0.000	4.485
CYP2T1P	0.198	0.175	0.138	0.611	0.413	0.425	2.839	1.505	0.009	2.034
STMN3	1.317	1.402	1.379	3.607	4.130	3.899	2.839	1.506	0.000	4.089
TOB1-AS1	0.263	0.117	0.276	0.611	0.620	0.638	2.849	1.510	0.001	2.842
PSCA	0.132	0.701	0.483	0.978	1.308	1.489	2.870	1.521	0.021	1.671
FAM78A	0.593	0.467	0.620	1.773	1.790	1.347	2.921	1.547	0.002	2.674
KLHDC9	1.120	0.701	1.241	2.262	3.924	2.765	2.923	1.548	0.019	1.714
HIST1H1C	6.059	6.659	8.480	19.747	19.962	22.329	2.927	1.549	0.000	3.608
PDZK1IP1	0.988	0.876	2.413	4.952	4.474	3.119	2.933	1.552	0.020	1.692
PKIA-AS1	0.198	0.234	0.207	0.550	0.826	0.496	2.935	1.553	0.016	1.795
HIST1H2A	0.263	0.467	0.620	1.100	1.308	1.559	2.936	1.554	0.007	2.181
RPL13AP2	0.593	0.234	0.276	1.284	1.033	0.922	2.938	1.555	0.010	1.987
FAM20C	0.724	0.292	0.552	1.467	1.652	1.489	2.939	1.555	0.002	2.731
ASPHD2	0.198	0.117	0.138	0.489	0.344	0.496	2.939	1.556	0.006	2.217
LTBP2	1.251	0.993	1.172	3.790	3.511	2.765	2.946	1.559	0.002	2.665
SDR42E1	0.000	0.175	0.207	0.428	0.275	0.425	2.954	1.563	0.038	1.417
PPL	0.856	1.402	1.310	3.974	3.098	3.473	2.955	1.563	0.002	2.800
SEMA6C	3.688	3.096	2.482	10.699	7.021	9.711	2.960	1.566	0.006	2.202

NLRC4	0.329	0.234	0.414	0.856	0.826	1.276	3.029	1.599	0.013	1.891
BMF	16.268	13.026	13.375	40.289	43.780	45.579	3.038	1.603	0.000	4.003
RND1	5.993	4.498	7.515	15.895	17.278	21.691	3.047	1.607	0.003	2.487
HIST1H2BI	3.359	2.629	3.378	9.660	11.977	7.018	3.060	1.613	0.011	1.940
RPS10	0.329	0.467	0.000	0.856	0.895	0.709	3.088	1.626	0.021	1.682
KRT7-AS	0.132	0.000	0.276	0.489	0.344	0.425	3.089	1.627	0.034	1.463
FBP1	0.461	0.350	0.620	1.345	0.964	2.127	3.097	1.631	0.046	1.334
SEMA4D	0.132	0.350	0.414	1.039	1.170	0.567	3.099	1.632	0.036	1.442
SATB1	0.329	0.584	0.620	1.834	1.859	1.063	3.100	1.632	0.018	1.749
SCARF1	1.120	0.701	0.207	2.323	2.409	1.559	3.103	1.634	0.020	1.706
HIST1H2B(	1.120	1.110	0.276	3.118	2.753	1.914	3.108	1.636	0.018	1.750
C6orf222	3.425	5.724	6.136	16.874	12.597	18.218	3.120	1.641	0.005	2.331
SAMD11	4.544	5.432	4.688	15.651	13.974	16.162	3.122	1.643	0.000	3.880
AHRR	0.922	0.876	1.034	2.507	3.235	3.119	3.128	1.645	0.001	3.018
SMG1P3	1.515	2.103	1.448	4.646	5.645	5.600	3.137	1.649	0.001	3.136
NPIPA8	0.395	0.292	0.138	0.795	1.239	0.567	3.152	1.656	0.049	1.314
WNT2B	0.329	0.292	0.138	0.734	0.895	0.780	3.172	1.665	0.002	2.719
ABTB1	13.436	10.398	11.927	39.983	38.617	34.946	3.175	1.667	0.000	3.927
SERPINI1	0.922	0.584	0.345	1.895	2.753	1.276	3.201	1.678	0.042	1.378
DGKA	0.659	0.759	0.345	1.895	1.996	1.772	3.213	1.684	0.001	3.118
LFNG	1.712	3.271	2.344	9.598	8.123	5.954	3.231	1.692	0.009	2.042
HIST2H2BI	1.449	1.577	1.930	4.769	6.884	4.395	3.238	1.695	0.009	2.028
SCARF2	0.329	0.584	0.207	1.467	1.239	0.922	3.238	1.695	0.012	1.906
ANKHD1	0.066	0.292	0.620	1.100	1.377	0.709	3.256	1.703	0.043	1.365
HIST1H2BI	6.652	6.776	3.861	19.258	18.792	18.288	3.259	1.704	0.000	3.710
PCDHGB9P	0.132	0.117	0.000	0.183	0.344	0.284	3.263	1.706	0.040	1.395
LRRC56	0.856	1.285	1.241	3.729	3.579	3.757	3.272	1.710	0.000	4.196
HIST1H2A(	0.527	0.993	0.896	3.179	2.340	2.410	3.282	1.715	0.004	2.423
ZDHHC1	0.659	0.234	0.207	1.528	0.826	1.276	3.303	1.724	0.029	1.542
MIR514B	0.329	0.000	0.138	0.489	0.413	0.709	3.448	1.786	0.043	1.366
SPDYA	0.527	0.643	0.000	0.978	1.790	1.276	3.458	1.790	0.036	1.443
KCND1	0.132	0.467	1.310	2.201	2.065	2.339	3.460	1.791	0.012	1.918
SYNGR3	0.856	1.051	0.138	3.118	2.616	1.347	3.461	1.791	0.048	1.319
HR	5.335	6.192	5.240	17.607	18.930	21.620	3.469	1.794	0.000	3.460
HAMP	0.461	0.350	0.000	0.734	1.308	0.780	3.477	1.798	0.044	1.357
RCAN2	0.461	0.643	0.138	1.956	1.377	0.992	3.484	1.801	0.031	1.502
OAZ3	0.066	0.409	0.207	0.917	0.826	0.638	3.493	1.805	0.012	1.928
SSTR5	0.856	0.058	0.965	2.629	2.478	1.489	3.509	1.811	0.026	1.578
ITGB8	0.132	0.701	0.965	2.262	1.859	2.197	3.514	1.813	0.005	2.262
AMIGO1	0.000	0.584	0.207	1.039	0.620	1.134	3.531	1.820	0.046	1.340
RAB3A	0.263	0.117	0.207	0.795	0.895	0.425	3.602	1.849	0.027	1.571
CD160	0.066	0.818	0.138	1.039	1.446	1.205	3.612	1.853	0.029	1.537
F10	0.198	0.350	0.207	0.978	0.757	0.992	3.613	1.853	0.002	2.714
KIAA1324	0.461	0.000	0.207	0.611	0.688	1.134	3.644	1.866	0.049	1.310
C1orf186	0.132	0.292	0.000	0.428	0.413	0.709	3.657	1.871	0.043	1.369
ADAMTS13	1.185	1.343	0.689	3.974	4.543	3.332	3.681	1.880	0.002	2.697
GAPDHP63	0.132	0.117	0.000	0.428	0.207	0.284	3.693	1.885	0.044	1.353
ACOT11	0.856	1.636	0.965	4.218	5.232	3.332	3.697	1.886	0.007	2.179
REPS2	0.527	0.935	0.414	2.507	2.409	2.056	3.718	1.894	0.001	2.902
MATN2	0.659	0.526	0.276	1.773	1.859	1.843	3.749	1.907	0.000	3.502
TRAM1L1	0.263	0.467	0.000	1.162	0.964	0.638	3.781	1.919	0.029	1.533
AGAP7P	0.066	0.058	0.069	0.245	0.207	0.284	3.802	1.927	0.001	2.886
SERPINB4	0.461	0.818	0.000	1.834	1.583	1.489	3.836	1.940	0.009	2.026
C19orf57	0.593	0.292	0.000	1.100	0.895	1.418	3.857	1.948	0.021	1.674
IL9RP3	0.329	0.117	0.000	0.672	0.413	0.638	3.863	1.950	0.028	1.554
RNU6-116	0.198	0.292	0.000	0.611	0.826	0.496	3.949	1.981	0.020	1.689
ITPR1-AS	0.132	0.234	0.000	0.611	0.275	0.567	3.979	1.992	0.044	1.354
UGT2B15	0.000	0.584	0.000	0.917	0.826	0.638	4.076	2.027	0.047	1.327
ADAM11	0.527	0.117	0.000	0.978	0.620	1.063	4.134	2.047	0.033	1.485
FRMPD2B	0.066	0.234	0.069	0.611	0.275	0.638	4.138	2.049	0.041	1.391
GPT	0.000	0.000	0.483	0.672	0.688	0.638	4.142	2.050	0.035	1.453
ZNF35	0.132	0.175	0.345	0.856	1.101	0.780	4.200	2.070	0.004	2.398
ADAMTS10	0.000	0.058	0.276	0.428	0.482	0.496	4.207	2.073	0.014	1.840
GHRL	0.132	0.058	0.000	0.245	0.344	0.213	4.215	2.075	0.021	1.684
RELN	0.263	0.234	0.207	0.795	0.895	1.347	4.314	2.109	0.010	1.985
FAM151B	0.198	0.234	0.138	0.856	1.033	0.567	4.315	2.109	0.010	1.979
RIPOR2	0.198	0.584	0.000	0.734	1.377	1.276	4.332	2.115	0.030	1.523
CHRNB4	0.132	0.175	0.000	0.428	0.620	0.284	4.336	2.116	0.037	1.435
SPDYE18	0.198	0.175	0.069	0.672	0.482	0.780	4.378	2.130	0.007	2.185
FOSB	0.461	0.759	0.896	3.057	2.891	3.402	4.418	2.143	0.000	3.583
GACAT2	0.066	0.058	1.103	2.079	1.583	1.772	4.427	2.146	0.020	1.693
USP2-AS1	0.000	0.350	0.069	0.550	0.482	0.851	4.489	2.166	0.035	1.453
FAM157B	0.000	0.117	0.000	0.183	0.207	0.142	4.551	2.186	0.033	1.480
CYP1A1	0.724	0.409	0.620	2.384	3.442	2.268	4.615	2.206	0.005	2.272
BIK	1.120	0.993	0.827	4.646	4.130	4.820	4.625	2.209	0.000	4.036
TMEM212	0.132	0.467	0.207	1.039	1.583	1.134	4.662	2.221	0.007	2.130
HIST1H2B(	1.976	0.759	0.552	6.603	5.025	3.757	4.681	2.227	0.013	1.902
IGFL2-AS	0.066	0.117	0.207	0.917	0.344	0.567	4.694	2.231	0.049	1.308
CD7	0.000	0.117	0.207	0.550	0.620	0.354	4.709	2.235	0.016	1.801
C2	0.395	0.117	0.000	0.611	0.826	0.992	4.746	2.247	0.017	1.782
SCTR	0.329	0.175	0.000	0.672	1.170	0.567	4.776	2.256	0.038	1.416
GRAP	0.066	0.058	0.000	0.183	0.275	0.142	4.832	2.273	0.024	1.627
HIST2H2BI	4.017	5.608	5.033	22.437	25.607	22.896	4.840	2.275	0.000	4.170
PCDHGA7	0.198	0.234	0.000	0.550	0.551	0.992	4.854	2.279	0.028	1.553

HIST1H2B	0.132	0.526	0.414	1.895	1.996	1.347	4.891	2.290	0.004	2.398
SAMMSON	0.198	0.000	0.000	0.428	0.207	0.354	5.005	2.323	0.046	1.333
ABHD1	0.066	0.000	0.000	0.122	0.138	0.071	5.023	2.329	0.042	1.382
LY6G6C	0.066	0.000	0.000	0.122	0.069	0.142	5.054	2.338	0.045	1.345
TINCR	0.000	0.350	0.000	0.795	0.482	0.496	5.058	2.339	0.038	1.423
HIST1H3E	0.659	0.350	0.138	1.712	2.065	2.056	5.085	2.346	0.001	2.917
AKAP3	0.198	0.058	0.207	0.611	0.964	0.780	5.088	2.347	0.005	2.303
RPL5P23	0.000	0.117	0.000	0.245	0.138	0.213	5.092	2.348	0.034	1.472
DPY19L1P	0.066	0.234	0.069	0.489	0.688	0.709	5.119	2.356	0.005	2.319
SSTR5-AS	0.395	0.000	0.552	1.467	1.583	1.843	5.169	2.370	0.003	2.573
MIR4479	0.000	0.350	0.000	0.489	0.620	0.709	5.186	2.375	0.021	1.671
MIRLET7D	0.066	0.234	0.000	0.367	0.413	0.780	5.207	2.380	0.047	1.328
DIO3OS	0.000	0.467	0.000	0.734	0.757	0.992	5.314	2.410	0.019	1.723
COL11A2	0.066	0.175	0.207	0.917	0.688	0.780	5.325	2.413	0.001	2.914
ANKRD1	0.527	0.292	0.689	2.629	2.547	2.906	5.358	2.422	0.000	3.799
ARHGAP30	0.000	0.175	0.069	0.306	0.482	0.567	5.548	2.472	0.016	1.796
NT5DC4	0.000	0.175	0.138	0.856	0.413	0.496	5.637	2.495	0.030	1.530
PRRT1B	0.000	0.526	0.000	1.100	1.101	0.780	5.671	2.504	0.016	1.787
CNTD1	0.198	0.058	0.138	0.978	0.482	0.780	5.686	2.508	0.015	1.831
DLL4	0.000	0.175	0.000	0.245	0.482	0.284	5.763	2.527	0.041	1.383
EIF4EP1	0.066	0.175	0.000	0.489	0.482	0.425	5.791	2.534	0.002	2.661
DNAAF3	0.198	0.117	0.138	1.162	0.826	0.638	5.805	2.537	0.010	2.022
HNRNPA1P	0.000	0.000	0.069	0.122	0.138	0.142	5.827	2.543	0.009	2.023
RPS3P2	0.000	0.058	0.000	0.061	0.138	0.142	5.831	2.544	0.045	1.347
TMEM47	0.066	0.175	0.000	0.306	0.688	0.425	5.887	2.558	0.034	1.469
KRT18P31	0.000	0.058	0.069	0.122	0.275	0.354	5.905	2.562	0.043	1.362
SPTBN5	0.066	0.175	0.207	0.978	0.826	0.851	5.927	2.567	0.000	3.495
CYP2A6	0.000	0.058	0.138	0.550	0.344	0.284	6.001	2.585	0.022	1.656
C1orf228	0.000	0.175	0.000	0.428	0.344	0.284	6.024	2.591	0.015	1.822
LEPR	0.263	0.350	0.138	1.528	1.308	1.701	6.035	2.593	0.001	3.207
ZBTB12BP	0.066	0.000	0.000	0.122	0.138	0.142	6.099	2.609	0.008	2.102
SHF	0.000	0.000	0.207	0.489	0.551	0.284	6.398	2.678	0.025	1.606
AGAP10P	0.198	0.058	0.000	0.489	0.344	0.851	6.578	2.718	0.042	1.375
HIST1H3H	0.000	0.058	0.620	1.895	1.514	1.347	7.006	2.809	0.006	2.219
CEACAM5	0.198	0.058	0.000	0.428	0.620	0.780	7.138	2.835	0.011	1.953
DDR2	0.066	0.000	0.138	0.611	0.207	0.638	7.145	2.837	0.045	1.345
IL34	0.263	0.000	0.000	0.856	0.551	0.496	7.223	2.853	0.018	1.734
MROH2A	0.000	0.117	0.000	0.245	0.275	0.354	7.484	2.904	0.008	2.114
RAB40A	0.132	0.058	0.000	0.550	0.620	0.284	7.643	2.934	0.018	1.739
HIST1H2B	0.593	0.409	0.000	2.323	2.409	3.048	7.768	2.958	0.001	2.846
ZNF568	0.329	0.000	0.000	0.428	1.033	1.134	7.879	2.978	0.037	1.427
SYT15	0.000	0.117	0.000	0.306	0.413	0.213	7.972	2.995	0.018	1.753
TESK2	0.329	0.234	0.414	2.935	2.822	2.056	7.999	3.000	0.001	2.900
RANBP3L	0.000	0.058	0.000	0.122	0.138	0.213	8.091	3.016	0.015	1.813
HIST1H4E	0.000	0.292	0.000	0.978	0.895	0.496	8.112	3.020	0.018	1.754
ACRBP	0.000	0.058	0.138	0.367	0.620	0.638	8.275	3.049	0.008	2.110
PSD	0.000	0.117	0.000	0.306	0.344	0.354	8.596	3.104	0.002	2.682
PRR34	0.000	0.117	0.000	0.306	0.275	0.425	8.614	3.107	0.008	2.105
TMEM191B	0.066	0.117	0.000	0.672	0.275	0.638	8.680	3.118	0.024	1.627
DDX18P1	0.066	0.000	0.000	0.306	0.138	0.142	8.884	3.151	0.044	1.359
GDF9	0.066	0.000	0.000	0.306	0.138	0.142	8.884	3.151	0.044	1.359
BMS1P7	0.000	0.058	0.000	0.245	0.138	0.142	8.970	3.165	0.018	1.748
CD226	0.000	0.000	0.138	0.550	0.413	0.284	9.042	3.177	0.015	1.836
CSNK1G2-	0.000	0.058	0.000	0.183	0.138	0.213	9.137	3.192	0.006	2.250
LDLRAD2	0.066	0.000	0.000	0.122	0.207	0.284	9.297	3.217	0.024	1.619
LHX2	0.066	0.000	0.000	0.122	0.207	0.284	9.297	3.217	0.024	1.619
PRSS27	0.066	0.058	0.000	0.672	0.275	0.284	9.909	3.309	0.050	1.303
ACP4	0.000	0.058	0.000	0.306	0.138	0.142	10.017	3.324	0.040	1.396
NHLRC4	0.066	0.000	0.000	0.122	0.207	0.354	10.374	3.375	0.045	1.349
CHRNA10	0.066	0.000	0.000	0.183	0.344	0.213	11.240	3.491	0.014	1.848
TBC1D10C	0.066	0.000	0.000	0.183	0.275	0.284	11.271	3.495	0.004	2.357
RPS11P6	0.000	0.117	0.000	0.489	0.275	0.567	11.397	3.511	0.013	1.877
TFCP2L1	0.000	0.058	0.000	0.122	0.344	0.213	11.626	3.539	0.037	1.430
GJC2	0.132	0.000	0.000	0.428	0.688	0.567	12.780	3.676	0.004	2.394
ARHGAP44	0.000	0.058	0.000	0.122	0.275	0.354	12.875	3.686	0.031	1.509
SPINT1-A	0.066	0.000	0.000	0.367	0.207	0.284	13.010	3.702	0.007	2.170
SLC17A7	0.066	0.000	0.000	0.245	0.275	0.354	13.275	3.731	0.002	2.621
KCNS1	0.066	0.000	0.000	0.122	0.413	0.354	13.509	3.756	0.040	1.400
ADAMTSL2	0.132	0.117	0.069	1.467	1.996	0.851	13.588	3.764	0.016	1.799
LBX1	0.066	0.000	0.000	0.306	0.207	0.425	14.235	3.831	0.012	1.912
C6orf99	0.000	0.058	0.000	0.245	0.413	0.213	14.898	3.897	0.014	1.847
SARM1	0.000	0.058	0.000	0.306	0.207	0.425	16.049	4.004	0.011	1.941
ABHD17AP	0.000	0.058	0.000	0.245	0.275	0.496	17.395	4.121	0.017	1.760
RFTN2	0.066	0.000	0.000	0.306	0.482	0.425	18.415	4.203	0.002	2.608
CLEC18B	0.000	0.058	0.000	0.367	0.275	0.496	19.488	4.285	0.006	2.238
SLC31A2	0.066	0.000	0.000	0.367	0.688	0.354	21.403	4.420	0.016	1.799
MIR4470	0.000	0.058	0.000	0.428	1.170	0.709	39.495	5.304	0.026	1.584
